# A scalable pipeline for designing reconfigurable organisms

**DOI:** 10.1073/pnas.1910837117

**Published:** 2020-01-13

**Authors:** Sam Kriegman, Douglas Blackiston, Michael Levin, Josh Bongard

**Affiliations:** ^a^Department of Computer Science, University of Vermont, Burlington, VT 05405;; ^b^Department of Biology, Tufts University, Medford, MA 02153;; ^c^Allen Discovery Center, Tufts University, Medford, MA 02153;; ^d^Wyss Institute for Biologically Inspired Engineering, Harvard University, Boston, MA 02115

**Keywords:** evolutionary computation, artificial life, bioengineering

## Abstract

Living systems are more robust, diverse, complex, and supportive of human life than any technology yet created. However, our ability to create novel lifeforms is currently limited to varying existing organisms or bioengineering organoids in vitro. Here we show a scalable pipeline for creating functional novel lifeforms: AI methods automatically design diverse candidate lifeforms in silico to perform some desired function, and transferable designs are then created using a cell-based construction toolkit to realize living systems with the predicted behaviors. Although some steps in this pipeline still require manual intervention, complete automation in future would pave the way to designing and deploying unique, bespoke living systems for a wide range of functions.

## Significance

Most technologies are made from steel, concrete, chemicals, and plastics, which degrade over time and can produce harmful ecological and health side effects. It would thus be useful to build technologies using self-renewing and biocompatible materials, of which the ideal candidates are living systems themselves. Thus, we here present a method that designs completely biological machines from the ground up: computers automatically design new machines in simulation, and the best designs are then built by combining together different biological tissues. This suggests others may use this approach to design a variety of living machines to safely deliver drugs inside the human body, help with environmental remediation, or further broaden our understanding of the diverse forms and functions life may adopt.

Most modern technologies are constructed from synthetic rather than living materials because the former have proved easier to design, manufacture, and maintain; living systems exhibit robustness of structure and function and thus tend to resist adopting the new behaviors imposed on them. However, if living systems could be continuously and rapidly designed ab initio and deployed to serve novel functions, their innate ability to resist entropy might enable them to far surpass the useful lifetimes of our strongest yet static technologies. As examples of this resistance, embryonic development and regeneration reveal remarkable plasticity, enabling cells or whole organ systems to self-organize adaptive functionality despite drastic deformation (1, 2). Exploiting the computational capacity of cells to function in novel configurations suggests the possibility of creating synthetic morphology that achieves complex novel anatomies via the benefits of both emergence and guided self-assembly (3).

Currently, there are several methods underway to design and build bespoke living systems. Single-cell organisms have been modified by refactored genomes, but such methods are not yet scalable to rational control of multicellular shape or behavior (4). Synthetic organoids can be made by exposing cells to specific culture conditions but very limited control is available over their structure (and thus function) because the outcome is largely emergent and not under the experimenter’s control (5). Conversely, bioengineering efforts with 3D scaffolds provide improved control (6–8), but the inability to predict behavioral impacts of arbitrary biological construction has restricted assembly to biological machines that resemble existing organisms, rather than discovering novel forms through automatic design.

Meanwhile, advances in computational search and 3D printing have yielded scalable methods for designing and training machines in silico (9, 10) and then manufacturing physical instances of them (11–13). Most of these approaches employ an evolutionary search method (14) that, unlike learning methods, enables the design of the machine’s physical structure along with its behavior. These evolutionary design methods continually generate diverse solutions to a given problem, which proves useful as some designs can be instantiated physically better than others. Moreover, they are agnostic to the kind of artifact being designed and the function it should provide: the same evolutionary algorithm can be reconfigured to design drugs (15), autonomous machines (11, 13), metamaterials (16), or architecture (17).

**Fig. 1.**
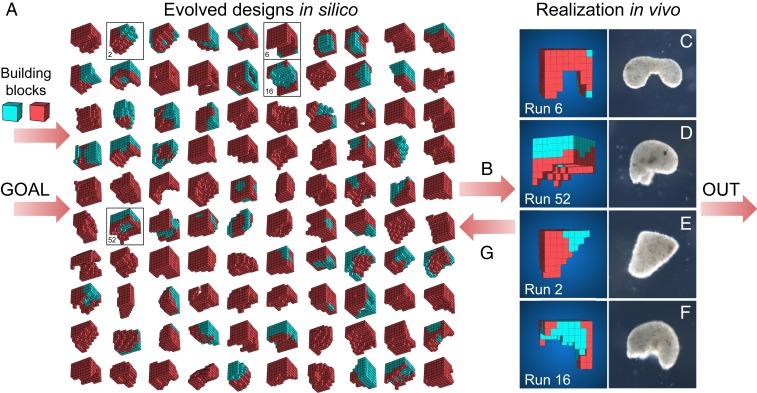
Designing and manufacturing reconfigurable organisms. A behavioral goal (e.g., maximize displacement), along with structural building blocks [here, contractile (red) and passive (cyan) voxels], are supplied to an evolutionary algorithm. The algorithm evolves an initially random population and returns the best design that was found. The algorithm is rerun 99 times starting with different random populations, generating a diversity of performant designs in silico (*A*; *SI Appendix*, section S5). Performant designs are then filtered by their robustness to random phase modulation of their contractile cells (*B*; *SI Appendix*, section S7), constructed in vivo using developing *Xenopus* cardiomyocyte and epidermal cell progenitors (*C–F*; *SI Appendix*, section S8), and placed on the surface of a Petri dish where their behavior is observed and compared to the design’s predicted behavior (*SI Appendix*, section S9). Discrepancies between in silico and in vivo behavior are returned to the evolutionary algorithm in the form of constraints on the kinds of designs that can evolve during subsequent design–manufacture cycles (*G*; *SI Appendix*, section S6). Concurrently, tissue layering and shaping techniques are modified such that realized living systems behave more like their virtual model (*SI Appendix*, section S8).

Here, we demonstrate a scalable approach for designing living systems in silico using an evolutionary algorithm, and we show how the evolved designs can be rapidly manufactured using a cell-based construction toolkit. The approach is organized as a linear pipeline that takes as input a description of the biological building blocks to be used and the desired behavior the manufactured system should exhibit (Fig. 1). The pipeline continuously outputs performant living systems that embody that behavior in different ways. The resulting living systems are novel aggregates of cells that yield novel functions: above the cellular level, they bear little resemblance to existing organs or organisms.

## Results

**Fig. 2.**
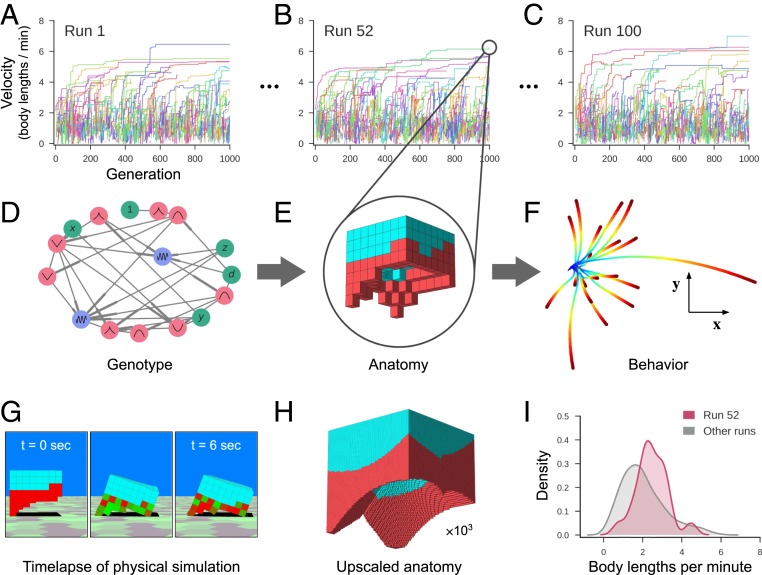
Designing reconfigurable organisms. For a given goal, 100 independent evolutionary trials were conducted in silico (*A–C*). Each colored line represents the velocity of the fastest-moving design within its clade. Each genome (*D*) dictates anatomy and behavior by determining where and how voxels are combined, and whether they are passive (cyan) or contractile (red; *E*). Genomes simulate a developmental process and are described in more detail in *SI Appendix*, section S4. The differing behavioral traces produced by a design (*F*) are a result of randomly perturbing the actuation of each contractile cell during each evaluation period. The behavioral traces all originate from the same position (blue) but diverge over time until their final destination (red). (*G*) During one evaluation period, after settling under gravity for 1 s, compressed and expanded contractile voxels are shown in red and green, respectively. Because the genotype is scale-free, the anatomical resolution of any design can be increased (*H*) while preserving geometry (but not necessarily behavior). When all evolutionary trials complete, the most performant design from each trial is extracted (*I*). The robust design passed to the next stage of the pipeline moves, on average, more rapidly (red curve) than the average speed of the other 99 designs (gray curve).

The pipeline is organized as a sequence of generators and filters (*SI Appendix*, Fig. S1). The first generator is an evolutionary algorithm that discovers different ways of combining the biological building blocks together to realize the desired behavior. A population of random designs are first created. Then, each design is simulated in a physics-based virtual environment and automatically assigned a performance score. Less-performant designs are deleted and overwritten by randomly modified copies of more-performant designs. Repeating this process yields populations of performant and diverse designs (Fig. 2).

As there are likely to be many differences between the simulated and targeted physical environments, performant designs are passed through a robustness filter which only allows passage of designs that sustain the desired behavior in the face of noise (*SI Appendix*, section S7). Previous work has shown that noise resistance in simulation is a simple and effective predictor of whether a design will maintain its behavior when instantiated physically (18).

The surviving noise-resistant designs are then passed through a build filter (*SI Appendix*, Fig. S4) which removes designs that are not suitable for the current build method (*SI Appendix*, Fig. S6) or unlikely to scale to more complex tasks in future deployments. The manufacturability of a design depends on the minimal concavity size that will persist in aggregations of developing stem cells, which tend to close small gaps in their collective geometry (*SI Appendix*, Fig. S7). The scalability of a design depends on its proportion of passive tissue, which provides space for future organ systems or payloads (*SI Appendix*, Fig. S13).

The designs that successfully pass through the build filter are then built out of living tissues. Pluripotent stem cells are first harvested from blastula stage *Xenopus laevis* embryos, dissociated, and pooled to achieve the desired number of cells. Following an incubation period, the aggregated tissue is then manually shaped by subtraction using a combination of microsurgery forceps and a 13-μm wire tip cautery electrode, producing a biological approximation of the simulated design. Further, contractile tissue can be layered into the organism through the harvesting and embedding of *Xenopus* cardiac progenitor cells, an embryonically derived cell type which naturally develops into cardiomyocytes (heart muscle) and produces contractile waves at specific locations in the resultant shaped form (*SI Appendix*, Fig. S6).

**Fig. 3.**
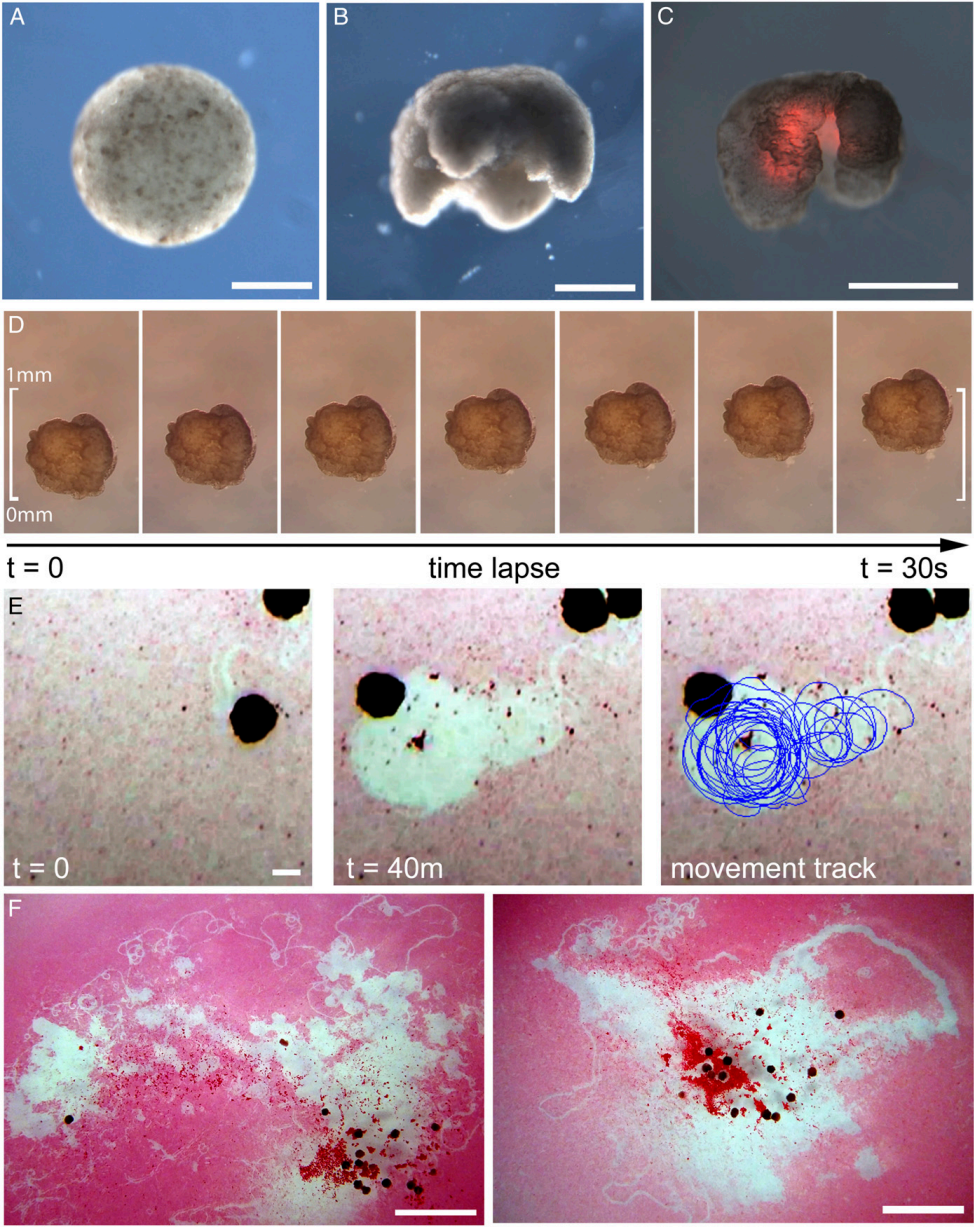
Manufacturing reconfigurable organisms. (*A*) Aggregation of pluripotent blastula cells harvested from *X. laevis* embryos. (*B*) Shaping results in 3D representations of the evolved in silico designs. (*C*) Layering of cardiac progenitor cells results in contractile cardiomyocyte tissue at specific locations, visualized by red fluorescent lineage tracer. (*D*) Time-lapse imaging of self-locomotion in an aqueous environment. (*E*) Emergent behavior of debris aggregation by an individual within the environment and (*F*) by groups of reconfigurable organisms over a 24-h period (*SI Appendix*, section S10.4). (Scale bars: 500 μm for *A–E* and 5 mm for *F*, respectively.)

**Fig. 4.**
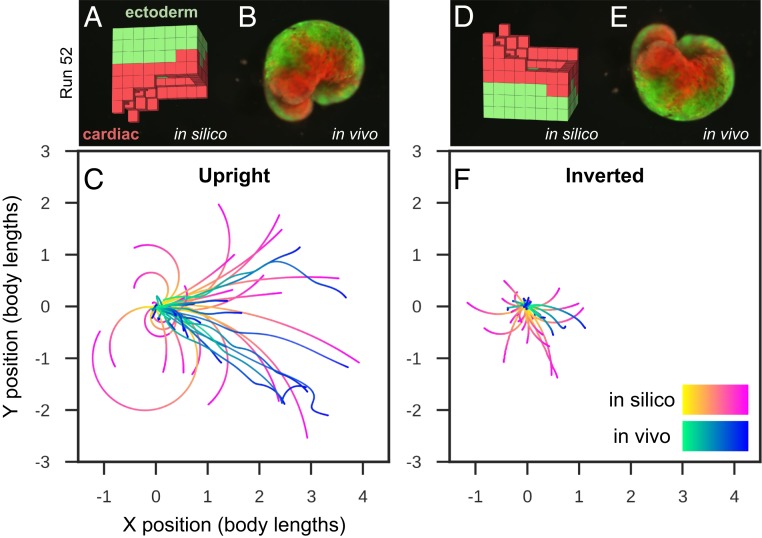
Transferal from silico to vivo. The first design selected for fabrication and specific hypothesis testing (*A*) was the most robust yet stable and energy-efficient configuration of passive (epidermis; green) and contractile (cardiac; red) tissues found by the evolutionary algorithm. The design was evaluated 25 times for 1 min of simulation time, resulting in 25 movement trajectories (pink curves in *C*). Six reconfigurable organisms were built which embodied this design (e.g., *B*) (*SI Appendix*, section S9). Three were evaluated four times and the other three were evaluated five times for 10 min each (27 blue curves in *C*). The organisms’ direction of movement matched the design’s predicted direction of movement (*P* < 0.01; details in *SI Appendix*, section S9). To determine whether the organisms’ movement was a result of chance or due to the design’s evolved geometry and tissue placement, geometry and tissue distribution was altered by rotating the design 180° about its transverse plane (*D*) and evaluating it another 25 times in silico (pink curves in *F*). Each of the six organisms were likewise inverted (*E*): four were evaluated five times while the remaining two were only evaluated once (22 blue curves in *F*). Inverting the design significantly reduces its net displacement (*P* < 0.001), as did inverting the organisms (*P* < 0.0001).

The final product of this procedure is a living, 3D approximation of the evolved design, which possesses the ability to self-locomote and explore an aqueous environment for a period of days or weeks without additional nutrients. These organisms are then deployed into their physical environment, and resultant behavior, if any, is observed (Fig. 3). Behaviors are then compared against those predicted by their simulated counterparts to determine whether or how well behaviors transferred from silico to vivo (Fig. 4).

After several organisms have been deployed and observed, it is likely that they exhibit varying amounts of the desired behavior. Common patterns among the successful systems are distilled down into constraints and supplied back to the evolutionary algorithm, which now evolves designs that are not just performant but also conform to the constraints (*SI Appendix*, section S6). This increases the success likelihood of subsequent design-to-deployment attempts.

Reconfigurable organisms were evolved to exhibit four different behaviors: locomotion, object manipulation, object transport, and collective behavior (*SI Appendix*, section S10). To achieve this, the pipeline was employed four times.

### Locomotion.

To obtain a diverse population of designs, 100 independent trials of the evolutionary algorithm were conducted (Fig. 2 *A*–*C*), each starting from a different set of initial random designs. During each trial, designs were selected based on net displacement achieved during a 10-s period (with randomized, phase-modulated contraction, cycling at 2 Hz). Additional selection pressures were applied to maintain diversity by inducing competition within and between unique genetic lineages within each trial (19), yielding unique ecological dynamics (*SI Appendix*, section S5). The most fit designs at the end of each trial were extracted (Fig. 1*A*) and passed through the robustness and build filters (*SI Appendix*, Fig. S4). During this filtering process, buildable and scalable designs that retain rapid locomotion during random perturbations are selected for manufacture (Fig. 3 and *SI Appendix*, Fig. S6).

Cilia, which produce locomotion through metachronal waves (the generation of sequential and directional propagating waves, as opposed to synchronized beating), were not modeled in silico and were suppressed in vivo through embryonic microinjection of mRNA transcribing the Notch intracellular domain (Notch ICD) (20). Thus, any displacement results from contractile cardiac muscle tissue that pushes against the surface of the dish. This simplifies the simulation and its comparison to the realized organism. Trajectories of deciliated designs are compared in silico and in vivo, in two orientations (upright and inverted 180° about the transverse plane) thus isolating the impact of the designed morphology on the difference between predicted and realized behavior. For at least one design, the data suggest that the desired behavior successfully transferred when it was upright but not when inverted (Fig. 4). More specifically, the upright organisms’ direction of movement matched that of the in silico design under random perturbations (*P* < 0.01; details in *SI Appendix*, section S9), and inverting the design significantly reduced its net displacement both in silico (*P* < 0.001) and in vivo (*P* < 0.0001). This suggests that successful transference did not result by chance but rather was due to the design itself.

### Object Manipulation.

When the environment is strewn with particulate matter, motile designs spontaneously aggregate the external objects both in silico (*SI Appendix*, Fig. S10) and in vivo (Fig. 3*F* and *SI Appendix*, Fig. S11). More precise object manipulation can be selected for an explicit goal, such as specifying target areas from which debris should be cleared, or target objects to discard. The latter goal was implemented and primitive end-effectors evolved in simulation (*SI Appendix*, Fig. S12).

### Object Transport.

Some designs evolved for displacement reduced hydrodynamic drag (*SI Appendix*, section S6) via a hole through the center of their transverse plane. This more complex topology was realized in vivo (*SI Appendix*, Fig. S13) but was not layered with contractile tissue. In simulation, this emergent feature can be exapted as a pouch to store and transport objects. In a subsequent round of evolution, pouches were explicitly incorporated as a design constraint, and the new goal of maximizing the distance of the carried object was employed. This yielded evolved object transport in silico (*SI Appendix*, Fig. S13).

### Collective Behavior.

Multiple designs can be placed in the same environment, yielding collective behavior (21) (*SI Appendix*, Figs. S10 and S11). Several such behaviors predicted in silico were observed in vivo. For instance, two designs often collide, form a temporary mechanical bond, and orbit about each other for several revolutions before detaching along tangential trajectories (*SI Appendix*, Fig. S10). This phenomenon is more pronounced when cilia are not inhibited on the organisms: individuals frequently become entangled with their neighbors, often changing partners across an observation (Fig. 3*F* and *SI Appendix*, Fig. S11).

## Discussion

Although simulation and design of rigid structures and machines has been possible for some time, only recently has it become computationally tractable to simulate the combined behavior of arbitrary aggregates of soft components with differing material and actuation properties (22). As shown here, such fine-grained simulations can be embedded in evolutionary search methods to discover designs that can be instantiated in biological rather than artificial materials.

The resulting organisms embodied not only the structure (*SI Appendix*, Fig. S8) of evolved in silico designs but also their behavior (Fig. 4), despite modeling cardiomyocyte temporal coordination as random noise. As a side effect of selection pressure for locomotion, derandomizing morphologies evolved: evolutionary improvement occurred through changes in overall shape, and distribution of the passive and contractile cells, to collectively derandomize the global movement produced by the random actuation. In biology, such robustness to random noise is ubiquitous; one example is the ability of many species to adapt to wide ranges of diversity in cell size and number as starting points in their embryogenesis (23).

The behavioral competence of individual cells, and the propensity of cells to cooperate in groups, facilitate functional morphogenesis in novel circumstances. The lifeforms presented here, despite lacking nervous systems, following novel developmental trajectories, and being composed of materials from different tissues, nevertheless possess these self-organizing properties. These properties synergize with and support the behavior they were designed to exhibit. For instance, although signaling between cardiomyocytes was not enforced, emergent spontaneous coordination among the cardiac muscle cells produced coherent, phase-matched contractions which aided locomotion in the physically realized designs. Also, some of the designs, when combined, spontaneously and collectively aggregate detritus littered within their shared environment (Fig. 3*F* and *SI Appendix*, Fig. S11). Finally, reconfigurable organisms not only self-maintain their externally imposed configuration, but they also self-repair in the face of damage, such as automatically closing lacerations (*SI Appendix*, Fig. S9). Such spontaneous behavior cannot be expected from machines built with artificial materials unless that behavior was explicitly selected for during the design process (24).

This approach admits future generalization and automation because the generator-and-filter architecture enables modular addition, removal, or reorganization of elements in the pipeline for rapid design and deployment of new living systems for new tasks in new domains. For instance, a filter could be added which preemptively steers the evolutionary algorithm away from portions of the design space known to contain designs that cannot be realized physically (25). Or, inspired by the hierarchical organization of deep neural networks (26), individual designs output by one generator could become the building blocks input to the next generator, thus enabling hierarchical design and reuse of cellular assemblies, and assemblies of assemblies.

Beyond the applications reported here, the generality of this approach is as of yet unknown. But, advances in machine learning, soft body simulation, and bioprinting are likely to broaden the potential applications to which it may be put in the future. Applications could be numerous, given the ease of misexpressing novel proteins and synthetic biology pathways and computational circuits in *Xenopus* cells (27). Given their nontoxicity and self-limiting lifespan, they could serve as a novel vehicle for intelligent drug delivery (28) or internal surgery (29). If equipped to express signaling circuits and proteins for enzymatic, sensory (receptor), and mechanical deformation functions, they could seek out and digest toxic or waste products, or identify molecules of interest in environments physically inaccessible to robots. If equipped with reproductive systems (by exploiting endogenous regenerative mechanisms such as occurs in planarian fissioning), they may be capable of doing so at scale. In biomedical settings, one could envision such biobots (made from the patient’s own cells) removing plaque from artery walls, identifying cancer, or settling down to differentiate or control events in locations of disease. A beneficial safety feature of such constructions is that in the absence of specific metabolic engineering, they have a naturally limited lifespan.

These methods, reagents, and data extend the breadth of model organisms available for study by designing living systems that are as orthogonal as possible to existing species, yet capable of being built from existing cell types. By enabling a computationally guided interplay between emergent and designed processes, this platform facilitates studies of the relationship between genomes (in our case, wild-type *X. laevis*), the resulting body plan, and its behaviors in diverse environments. Thus, reconfigurable organisms could serve as a unique model system facilitating work in the evolution of multicellularity, exobiology, artificial life, basal cognition, and regenerative medicine. If equipped with electrically active cells and selected for cognitive or computational functions (30), such designed systems may similarly broaden our understanding of how intelligence can be instantiated in living as well as nonliving systems.

## Materials and Methods

### Evolutionary Design.

Designs (*SI Appendix*, section S2) were evolved inside a physics engine (*SI Appendix*, section S3) as reconfigurable aggregations of passive and contractile voxels (Fig. 1). On the first pass through the pipeline using the goal behavior of locomotion, we simulated designs on land and allowed the evolutionary process to finely tune their actuation. This resulted in highly performant but nontransferable designs (*SI Appendix*, Fig. S2) with powerful, bounding gaits that are not obtainable in vivo with the current build method (*SI Appendix*, section S8). These gaits were characterized by timeframes (on average, 47% of the gait cycle) in which no part of the in silico design was in contact with the simulated ground plane. In vivo, however, the deciliated organisms always kept part of their ventral surfaces in contact with the surface of the dish due to negative buoyancy.

These discrepancies were rectified by adding constraints into the pipeline in the form of adjustments to environmental and actuation settings, which were altered as follows. On the second pass, the fidelity of the simulated environment was increased by incorporating first-order hydrodynamics: the modified environment consisted of an infinite plane submerged in water, which was approximated by decreasing the coefficient of gravitational acceleration (increasing buoyancy) and applying a drag force to each voxel face on the design’s surface (*SI Appendix*, section S6).

Secondly, actuation was randomized: contractile cells were revised to have random phase offsets from a central pattern generator (a sine wave with frequency 2 Hz). More specifically, each voxel of a randomly configured design (one of which was injected into the population at each generation; *SI Appendix*, section S5) was assigned a random phase offset, which was held fixed in its descendants (the entire clade). Mutations switched each voxel to be either present or absent, and, if present, either passive or active (contractile), but the original phase offset, at every location in the workspace, was hardcoded. This reduced the dependence on precisely timed excitation, and promoted the discovery of more robust mechanical structures (*SI Appendix*, Fig. S3).

The behavior of designs generated on the second pass better matched the behavior of the actual living systems: on average, designs were in contact with the ground plane for 93.3% of their evaluation period, compared to just 52.7% on the first pass (*SI Appendix*, section S6).

### Robustness Filter.

The most performant designs (Fig. 1*A*) were sorted by their robustness to random perturbations in their actuation. Phase offsets stored in the genotype were mutated by adding a number that was drawn randomly from a normal distribution with mean zero and SD *s* = 0.4π (which is 40% of the −π/2 to π/2 range of valid phase-offset values). This hyperparameter was selected to be large enough to scramble the original phase-offset value without being so large as to push all mutations up against the ±π/2 bounds. Designs that maintained the highest average performance across this actuation noise were passed, one by one, in order of their robustness ranking, to the build filter.

### Build Filter.

The most robust designs are evaluated by their manufacturability under the current build method, which layers contiguous tissue regions sequentially (*SI Appendix*, Fig. S6). The minimal concavity was examined by producing organisms with progressively smaller shape deformations, then determining which persist across the lifespan of the organism, and which close due to tissue contraction, leading to loss of concavity. Preliminary work determined that concavities with a width of 100 µm or greater (12% of total body length) produced stable long-term deformations suitable for biological building (*SI Appendix*, Fig. S7).

Additionally, the build filter removes designs that are more than 50% muscle, in order to reserve sufficient design space to add specialized cells for purposes other than locomotion, including sensory input, metabolism, memory, biosensors, etc. Also, contractile tissue incurs a much higher metabolic cost compared to nonmuscle tissue (the human heart consumes ∼1 mM ATP per second; ref. 31). Thus, limiting this tissue type increases the total lifetime of transferred designs. The most robust designs that satisfy these selection criteria (*SI Appendix*, Fig. S4) are passed through the build filter to the next stage of the pipeline: the realizability generator.

### Realizability Generator.

Reconfigurable organisms were created using *Xenopus* embryos as donor tissue under methods approved by the Institutional Animal Care and Use Committee and Tufts University Department of Laboratory Animal Medicine under protocol number M2017-53.

Fertilized *X. laevis* eggs were reared in a 0.1×, pH 7.8, Marc’s Modified Ringers solution (MMR) using standard protocols and staged according to Nieuwkoop and Faber (32, 33). For shaping experiments, animal caps were manually cut at St. 9 using surgery forceps (Dumont, 11241–30 #4) and transferred to calcium- and magnesium-free medium for 5 min (50.3 mM NaCl, 0.7 mM KCl, 9.2 mM Na_2_HPO_4_, 0.9 mM KH_2_PO_4_, 2.4 mM NaHCO3, 1.0 mM edetic acid [EDTA], pH 7.3). The outer ectoderm layer was manually removed and discarded, while the inner layer was agitated until fully dissociated (cells are this stage are largely pluripotent, but differentiate into ectoderm without further intervention). Material from five animal caps was pooled and transferred to a welled dish containing 0.75× MMR. After 24 h at 14 °C, the spherical reaggregate was moved to a clean 1% agarose-coated dish containing 10 mL 0.75× MMR and 5 µl gentamicin (ThermoFisher Scientific, 15710072). Forty-eight hours after tissue reaggregation the resulting tissue (now fated to become specific epidermal cell lineages including ionocytes, small secretory cells, and goblet cells), was shaped using a combination of microsurgery forceps and a MC-2010 microcautery instrument with 13-μm wire electrodes (Protech International Inc., MC-2010, 13-Y1 wire tip cautery electrode). Tissue was reshaped as necessary for 3 h to create the desired anatomical outcome, after which it was moved to a clean 1% agarose-coated dish containing 10 mL 0.75× MMR and 5 µl gentamicin and raised at 14 °C.

For contractile movement experiments, cohorts of *Xenopus* embryos were microinjected with one of two synthetic mRNAs at the four-cell stage using standard protocols (32). mRNA for the fluorescent lineage tracer tdTomato (34) and the multiciliated cell inhibitor Notch ICD (20, 35) was synthesized using mMESSAGE transcription kits (ThermoFisher Scientific, AM1340). Injections were performed in 3% Ficoll solution using a pulled capillary to deliver 370 pg of mRNA for each transcript to all four cells. tdTomato microinjected embryos were reared for at 22 °C while Notch ICD injected embryos were reared at 14 °C. Twenty-four hours after injection, stage 10 Notch ICD injected embryos were moved to a 1% agarose-coated Petri dish containing 0.75× MMR, and animal caps were manually cut using surgery forceps as above. In addition, stage 23–24 tdTomato injected embryos were transferred to the same dish and the presumptive heart field was excised with the outer layer of ectoderm then removed and discarded. Presumptive heart tissue was then placed between two Notch ICD injected animal caps, and the three layers were allowed to heal for 1 h at 22 °C. Following healing, the tissue was moved to clean 1% agarose-coated dish containing 10 mL 0.75× MMR and 5 µl gentamicin and raised at 14 °C. For shaping, resultant tissue was sculpted as above using a combination of microsurgery forceps and a MC-2010 microcautery instrument.

### Transferability Filter.

All samples were imaged live in 0.75× MMR at 20 °C using a Nikon SMZ-1500 microscope equipped with both top and substage illumination. Still images were captured on a QImaging Retiga 2000R CCD camera and videos were captured using a Sony IMX234 at a sample rate of 30 frames per second. XY movement tracks were extracted for each run using Noldus Ethovision 14 software, and smoothed using a one-dimensional Gaussian filter (*SI Appendix*, section S9.1). The tdTomato lineage tracer was imaged using a standard tetramethylrhodamine isothiocyanate (TRITC) filter cube and fluorescent light source to verify cardiac muscle cell location, and GFPIII signal was imaged with a standard fluorescein isothiocyanate (FITC) filter cube to verify epidermal cell location (*SI Appendix*, section S9.2).

### Data Availability.

The source code necessary for reproducing the computational results reported in this paper can be found at GitHub (36).

## Supplementary Material

Supplementary File

Supplementary File

Supplementary File

## References

[1] Blackiston D. J., Levin M., Ectopic eyes outside the head in Xenopus tadpoles provide sensory data for light-mediated learning. J. Exp. Biol. 216, 1031–1040 (2013). 23447666 10.1242/jeb.074963 PMC3587383

[2] Vandenberg L. N., Adams D. S., Levin M., Normalized shape and location of perturbed craniofacial structures in the Xenopus tadpole reveal an innate ability to achieve correct morphology. Dev. Dyn. 241, 863–878 (2012). 22411736 10.1002/dvdy.23770 PMC3428595

[3] Kamm R. D., Perspective: The promise of multi-cellular engineered living systems. APL Bioeng 2, 040901 (2018). 31069321 10.1063/1.5038337 PMC6481725

[4] Hutchison C. A. III, Design and synthesis of a minimal bacterial genome. Science 351, aad6253 (2016). 27013737 10.1126/science.aad6253

[5] Sasai Y., Eiraku M., Suga H., In vitro organogenesis in three dimensions: Self-organising stem cells. Development 139, 4111–4121 (2012). 23093423 10.1242/dev.079590

[6] Park S. J., Phototactic guidance of a tissue-engineered soft-robotic ray. Science 353, 158–162 (2016). 27387948 10.1126/science.aaf4292 PMC5526330

[7] Tang-Schomer M. D., Bioengineered functional brain-like cortical tissue. Proc. Natl. Acad. Sci. U.S.A. 111, 13811–13816 (2014). 25114234 10.1073/pnas.1324214111 PMC4183301

[8] Nawroth J. C., A tissue-engineered jellyfish with biomimetic propulsion. Nat. Biotechnol. 30, 792–797 (2012). 22820316 10.1038/nbt.2269 PMC4026938

[9] Sims K., Evolving 3D morphology and behavior by competition. Artif. Life 1, 353–372 (1994).

[10] Cheney N., Bongard J., SunSpiral V., Lipson H., Scalable co-optimization of morphology and control in embodied machines. J. R. Soc. Interface 15, 20170937 (2018). 29899155 10.1098/rsif.2017.0937 PMC6030623

[11] Lipson H., Pollack J. B., Automatic design and manufacture of robotic lifeforms. Nature 406, 974–978 (2000). 10984047 10.1038/35023115

[12] Bongard J., Zykov V., Lipson H., Resilient machines through continuous self-modeling. Science 314, 1118–1121 (2006). 17110570 10.1126/science.1133687

[13] Cellucci D., MacCurdy R., Lipson H., Risi S., 1D printing of recyclable robots. IEEE Robot. Autom. Lett. 2, 1964–1971 (2017).

[14] Munk D. J., Vio G. A., Steven G. P., Topology and shape optimization methods using evolutionary algorithms: A review. Struct. Multidiscipl. Optim. 52, 613–631 (2015).

[15] Devi R. V., Sathya S. S., Coumar M. S., Evolutionary algorithms for de novo drug design–A survey. Appl. Soft Comput. 27, 543–552 (2015).

[16] Huntington M. D., Lauhon L. J., Odom T. W., Subwavelength lattice optics by evolutionary design. Nano Lett. 14, 7195–7200 (2014). 25380062 10.1021/nl5040573 PMC4264853

[17] Mueller C. T., Ochsendorf J. A., Combining structural performance and designer preferences in evolutionary design space exploration. Autom. Constr. 52, 70–82 (2015).

[18] Jakobi N., Evolutionary robotics and the radical envelope-of-noise hypothesis. Adapt. Behav. 6, 325–368 (1997).

[19] Schmidt M., Lipson H., “Age-fitness pareto optimization” in Genetic Programming Theory and Practice VIII (Springer, 2011), pp. 129–146.

[20] Deblandre G. A., Wettstein D. A., Koyano-Nakagawa N., Kintner C., A two-step mechanism generates the spacing pattern of the ciliated cells in the skin of Xenopus embryos. Development 126, 4715–4728 (1999). 10518489 10.1242/dev.126.21.4715

[21] Werfel J., Petersen K., Nagpal R., Designing collective behavior in a termite-inspired robot construction team. Science 343, 754–758 (2014). 24531967 10.1126/science.1245842

[22] Hiller J., Lipson H., Dynamic simulation of soft multimaterial 3d-printed objects. Soft Robot. 1, 88–101 (2014).

[23] Cooke J., Scale of body pattern adjusts to available cell number in amphibian embryos. Nature 290, 775–778 (1981). 7219562 10.1038/290775a0

[24] Kriegman S., Walker S., Shah D., Levin M., Kramer-Bottiglio R., Bongard J., Automated shapeshifting for function recovery in damaged robots. Proc. Rob. Sci. Syst. (2019).

[25] Koos S., Mouret J. B., Doncieux S., The transferability approach: Crossing the reality gap in evolutionary robotics. IEEE Trans. Evol. Comput. 17, 122–145 (2013).

[26] Zeiler M. D., Fergus R., “Visualizing and understanding convolutional networks” in Proceedings of the European Conference on Computer Vision, Fleet D., Pajdla T., Schiele B., Tuytelaars T., Eds. (Springer, 2014), pp. 818–833.

[27] Toda S., Blauch L. R., Tang S. K. Y., Morsut L., Lim W. A., Programming self-organizing multicellular structures with synthetic cell-cell signaling. Science 361, 156–162 (2018). 29853554 10.1126/science.aat0271 PMC6492944

[28] Patra D., Intelligent, self-powered, drug delivery systems. Nanoscale 5, 1273–1283 (2013). 23166050 10.1039/c2nr32600k

[29] Li J., Esteban-Fernández de Ávila B., Gao W., Zhang L., Wang J., Micro/nanorobots for biomedicine: Delivery, surgery, sensing, and detoxification. Sci. Rob. 2, eaam6431 (2017). 10.1126/scirobotics.aam6431 PMC6759331 31552379

[30] Baluška F., Levin M., On having no head: Cognition throughout biological systems. Front. Psychol. 7, 902 (2016). 27445884 10.3389/fpsyg.2016.00902 PMC4914563

[31] Piquereau J., Ventura-Clapier R., Maturation of cardiac energy metabolism during perinatal development. Front. Physiol. 9, 959 (2018). 30072919 10.3389/fphys.2018.00959 PMC6060230

[32] Nieuwkoop P. D., Faber J., Normal Table of Xenopus laevis (Daudin) (North Holland Publishing Co., Amsterdam, 1956).

[33] Sive H. L., Grainger R. M., Harland R. M., Early Development of Xenopus laevis (CSHL Press, 2000).

[34] Waldner C., Roose M., Ryffel G. U., Red fluorescent Xenopus laevis: A new tool for grafting analysis. BMC Dev. Biol. 9, 37 (2009). 19549299 10.1186/1471-213X-9-37 PMC2706234

[35] Beck C. W., Slack J. M., A developmental pathway controlling outgrowth of the Xenopus tail bud. Development 126, 1611–1620 (1999). 10079224 10.1242/dev.126.8.1611

[36] Kriegman S., Blackiston D., Levin M., Bongard J., Data from “A scalable pipeline for designing reconfigurable organisms.” GitHub. https://github.com/skriegman/reconfigurable_organisms. Deposited 2 October 2019. 10.1073/pnas.1910837117 PMC6994979 31932426

