# Supplementary material for: A scalable pipeline for designing reconfigurable organisms
Source: Proc Natl Acad Sci U S A. 2020 Jan 13;117(4):1853–9. doi: 10.1073/pnas.1910837117 (PMC6994979; doi:10.1073/pnas.1910837117)
Supplement: Supplementary File [file pnas.1910837117.sapp.pdf]

Supplementary information for

A scalable pipeline for designing reconfigurable organisms

Sam Kriegman, Douglas Blackiston, Michael Levin, and Josh Bongard

www.pnas.org/cgi/doi/10.1073/pnas.1910837117

Table of contents:

List of Supplementary Videos 4

List of Supplementary Tables 4

List of Supplementary Figures 4

Supplementary Methods 5

1. The source code. 5
2. The design space. 5
3. The physics engine. 5
4. The encoding. 6
    - 4.1. Genotype networks. 6
    - 4.2. Morphological complexity. 7
5. Evolutionary design. 7
    - 5.1. The algorithm. 7
    - 5.2. Measuring performance. 8
    - 5.3. Runtime. 9
    - 5.4. Algorithm analysis. 9
    - 5.5. Comparison to a gradient-based approach. 9
6. Updating design constraints. 10
7. Filtering evolved designs for manufacture. 11
    - 7.1. The robustness filter. 11
    - 7.2. The build filter. 12
8. Manufacturing reconfigurable organisms 12
    - 8.1. Cilia-driven organisms. 12
    - 8.2. Cardiomyocyte-driven organisms. 13
9. Measuring transfer success. 13

9.1. Transferal of behavior. 13

9.2. Transferal of structure. 14

10. Applications. 16

10.1. Object manipulation. 16

10.2. Object transport. 16

10.3. Object expulsion. 17

10.4. Collective behavior. 17

11. Scaling the pipeline. 17

Supplementary References **19**

List of Supplementary Videos:

Video S1. Designing reconfigurable organisms. 20

Video S2. Manufacturing reconfigurable organisms. 21

List of Supplementary Tables:

Table S1. Biological reagents. 22

Table S2. Shaping tools. 23

Table S3. Imaging. 23

List of Supplementary Figures:

S1. Pipeline diagram. 24

S2. The fastest designs from the first pass of the pipeline for locomotion. 25

S3. The fastest designs from the second pass of the pipeline for locomotion. 26

S4. The design filters and selection criteria. 27

S5. The most robust design to pass through the filter. 28

S6. Manufacturing cardiomyocyte-driven reconfigurable organisms. 29

S7. Determining minimal concavity when shaping reconfigurable organisms. 30

S8. Quantifying the structural match. 31

S9. Automatic self-repair in reconfigurable organisms. 32

S10. Spontaneous collective behavior and particle aggregation *in silico*. 33

S11. Aggregation of carmine particles occurs only in the presence of organisms. 34

S12. Explicit selection for object manipulation *in silico*. 35

S13. From spontaneous to explicitly-optimized object transportation *in silico*. 36

S14. Object expulsion *in silico*. 37

S15. The encoding bias. 38

S16. Stochastic gradient descent directly applied to the design problem. 39

S17. Algorithm analysis. 40

S18. Scaling the pipeline. 41

Supplementary Methods

1. The source code: github.com/skriegman/reconfigurable_organisms

2. The design space.

This subsection gives bounds on the number of designs that can be built in a voxel-based workspace.

A distinct configuration of exactly N congruent cubes (voxels) connected face-to-face is known as a *polycube*. With N=2, there is just a single configuration: a 2-by-1 column (or dicube). But there are two perpendicular rotations: if one cube is resting on the ground, we can add the second on top or on the side.

Rotations of the same configuration can be treated as equivalent, or not. Polycubes are called "real" if equivalent under rotation, and "fixed" if not. With N=3, there are two real polycubes (tricubes), and 15 fixed. With N=4, there are eight real (tetracubes), and 86 fixed. As N increases, the number of polycubes grows exponentially. With N=16 cubes, for instance, there are on the order of 10^{10} real polycubes and 10^{12} fixed polycubes (36).

But this assumes that all cubes are identical. Here, the design space consists of polycubes composed of two cube types (passive and contractile), with any N, that fit inside an 8×8×7 bounding box. Many polycubes with N>7 won't fit inside this workspace. And with N=8×8×7=448, there is only one polycube geometry that fits, but there are still $2^{448} = 7.27\times10^{134}$ possible combinations of passive and contractile voxels that could be used to build the polycube.

Because the designs here are functional polycubes, their uniqueness depends on the behavioral goal. For locomotion in any direction away from the origin on a surface plane, designs can yield different behavior when inverted (Fig. 4), but the same behavior when the design is rotated about the vertical axis (just facing a different direction). Because there are three tissue options (none, passive, and contractile) at each point in the workspace, there are $3^{448} = 5.63\times10^{213}$ possible configurations, though some are isomorphic translations after reducing to a single (the largest) polycube.

3. The physics engine.

This subsection briefly describes the physics of the *in silico* environment. For more details, see (22).

Experiments were performed using the voxel-based physics engine Voxelyze (22). Voxels were connected to each other on a regular grid to form a contiguous geometry: a polycube. Interactions between adjacent voxels were modeled as flexible beams (critically damped; zeta=1) according to Euler–Bernoulli beam theory. A Coulomb friction model was applied to voxels in contact with the ground surface plane. Volumetric actuation resulting from contractile cells was simulated by oscillating the rest length between adjacent actuating voxels, in all three dimensions, when computing the elastic force between them. Additionally, a collision detection system ensured that the organisms did not self-penetrate. If a pair of surface voxels are detected to collide (intersect), a temporary beam (underdamped; zeta=0.8) was constructed between the two until the collision is resolved. A time step of 0.0032 seconds was used for numerical

integration. Each design was allowed to settle under gravity for 312 timesteps (one second) before an evaluation period of 3125 timesteps (10 seconds), resulting in a total simulation time of 11 seconds.

4. The encoding.

This subsection defines the genetic search space evolution operates in. Source code: github.com/skriegman/reconfigurable_organisms/blob/master/networks.py

4.1. *Genotype networks.* Each configuration is genetically encoding using a Compositional Pattern-Producing Network, or CPPN (37), that maps the spatial coordinates of a 3D cartesian lattice to a single value indicating whether there is material at that location, and, if so, whether it is a contractile or passive cell. The genotype encoding is thus scale-free: A given genotype network can be mapped to arbitrary resolution coordinate space (Figs. 2*H* and S18).

We chose this particular encoding because it tends to generate spatial regularities in structure, which are known to facilitate locomotion (38). In short, CPPNs are a special class of networks that use various activation (or interaction) functions that output regular patterns, such as sine waves and parabolas (instead of using only a single sigmoid or ramp function). CPPNs were originally proposed (37) as an abstraction of gene expression and embryonic development (rather than of neurons and brains), and we employ them as such here.

Input/output. The coordinates of each voxel are specified by their cartesian coordinates (x, y, z) and radial distances from the center of the lattice workspace (here, 8-by-8-by-7). These four coordinates (in addition to a bias term set equal to 1) are taken as input to the network. The inputs connect to (interact with) various regulatory and structural genes (vertices) by weighted edges (with real-valued scalar weights in the range -1 to +1) that multiply the input by the corresponding weight. Regulatory and structural genes sum all incoming weighted edges as input for their interaction function, which is here taken to be any of the following: sin(), abs(), square(), sqrt(abs()); and the negations of those four. The output expressions of regulatory genes are reweighted and passed onward to interact with additional regulatory genes (hidden nodes) or else with one of two structural genes (output nodes), whose output is thresholded at zero to determine (express) material presence and type, respectively. In some experiments (Figs. S2 and S14), an additional, independent CPPN was used to determine the phase-offset (of open-loop volumetric actuation) at each voxel.

Mutation. Both the architectures and weights of these networks are evolved: mutations add, modify or remove a single randomly-selected vertex or edge. Offspring are created by performing six kinds of mutations (add vertex/edge, remove vertex/edge, modify vertex/edge), each of which is applied with probability 1/6. If none are selected (which occurs with probability $(5/6)^6 = 0.33$), one of the six is randomly chosen and applied. If a mutation is neutral (i.e., the resulting phenotypic structure is unchanged), another mutation is applied; after 1500 unsuccessful attempts at a non-neutral mutation, the 1500 neutral mutations are accepted. After mutation, the network is pruned of any erroneous edges and vertices that are not connected to the main graph (details about the evolutionary algorithm can be found in §5.1).

Initialization, hyperparameters. Networks are initialized by fully connecting the input layer to the output, and normalizing the inputs (the coordinates of the workspace) to be between 0 and 1. Then, ten random vertices are added. Next, ten randomly-selected pairs of unconnected

vertices are attached by a new edge, the weight of which is drawn from a uniform distribution between -1 and 1. (If the new edge creates a cyclic graph, erase it and retry, 999 times. If 1000 failed attempts occur, terminate.) After these additions, five randomly selected edges are removed. Then, 100 edges are randomly selected (with replacement) and their weights are mutated (serially) by adding a value drawn from a normal distribution with mean zero and standard deviation, s=0.5, clipping the new weight to be between -1 and 1. (Neutral mutations are permitted during this initialization process.) Finally, 100 vertices are likewise selected with replacement, and their interaction functions are replaced by functions randomly chosen from the set {±sin(), ±abs(), ±square(), ±sqrt(abs())}. Hyperparameters were adopted from (24).

4.2. *Morphological complexity.* In this work, we have demonstrated the evolution of a CPPN encoding toward various geometries (Fig. S3) and topologies (Fig. S13) capable of open-loop locomotion and object manipulation in a simulated environment. However, these shapes tend to be relatively compact and simple, lacking more complex structures, such as branching limbs.

Previous work linked morphological complexity resulting from CPPN-genotypes with environmental complexity (39). We likewise observed the optimization of environment-specialized morphologies, such as the evolution of rudimentary end-effectors that hold an external object in place during its manipulation (Fig. S12).

To demonstrate the flexibility of the encoding to achieve more complex morphologies as dictated by the task environment, we optimized CPPNs for a single evolutionary run to generate passive structures that match target shapes (Fig. S15). To do so, we used the same evolutionary algorithm and hyperparameters (detailed below in §5) with a modified objective function: Performance was defined as the Hamming distance between binary matrices of the target and CPPN-output shapes (instead of locomotion velocity).

In a separate experiment in which designs were challenged to throw an object (§10.3), we successfully evolved functional limbs by including an objective to maximize the design's surface-area to volume ratio, in addition to an objective for thrown object distance (Fig. S14). It would be of interest to explore, in future work, the genetic encodings and behavioral selections pressures that indirectly select for complex organism geometries.

5. Evolutionary design.

This subsection outlines the evolutionary algorithm and its objective functions, analyzes the algorithm in smaller solution spaces, and compares evolution to a gradient-based approach.

5.1. *The algorithm.* A standard evolutionary algorithm was employed: Age-Fitness-Pareto Optimization, or AFPO (19). AFPO uses the concept of Pareto dominance and an objective of "age" (in addition to performance) intended to promote diversity among candidate designs and prevent premature convergence. "Age" is a clade-level attribute that counts the number of generations a clade has existed in the population: Each design can trace its ancestral roots back to a randomly configured, parentless individual, which was injected into the population, with age zero, at some previous generation. Thus the age roughly corresponds to the amount of search time spent in a particular area of design space.

One hundred independent evolutionary trials were conducted, each with a unique random seed (which is here set equal to the run number, 1-100), culminating in a unique *run champion*: the most performant design found, according to the goal function (Figs. S2 and S3).

Each trial is initialized with a population of 50 randomly-configured designs with age zero. Every generation, the population is first doubled by creating modified copies of each individual in the population (offspring have the same age as their parent). The age of each individual is then incremented by one. Next, an additional randomly-configured individual (with age zero) is injected into the population (which now consists of 101 designs). Finally, selection reduces the population down to its original size (50 designs) according to the two objectives of performance (maximized) and age (minimized). That is, starting with the youngest and most performant designs, which are by definition nondominated, successive Pareto fronts are kept in their entirety until doing so would overfill the population past its original size (50 designs), at which point designs are added stochastically with probability proportional to their performance. This process of random variation and directed selection is repeated for 1000 generations.

A third objective was used in addition to performance and age: number of contractile voxels (minimized). This additional objective was added to ensure organisms had sufficient non-functional volumes that could be replaced by future non-actuating building blocks which may be required for specific tasks, such as wavelength perception (opsin-like detection), or metabolic pathways necessary for nutrient uptake and consumption. In spite of this objective, none of the run champions from the first pass for locomotion utilized passive voxels (Fig. S2).

Similarity to other architecture search algorithms. As mutations not only tune the parameters of an existing (parent) network, but can also add and remove genotype network structure (edges and vertices), the evolutionary algorithm is performing what is known as "architecture search" (40). Many evolutionary approaches to architecture search exist, such as NEAT (NeuroEvolution of Augmenting Topologies; (41)). However, NEAT evolves artificial neural networks, whereas we are evolving bodyplans. More specifically, we are evolving CPPNs (genotype; §4.1) that encode bodyplans (phenotype). HyperNEAT (Hybercube-based NeuroEvolution of Augmenting Topologies; (42)) is an extension of the NEAT method which evolves CPPNs that typically, as the name suggests, encode neural network connectivity patterns. However, HyperNEAT has also been used to evolve CPPNs that encode soft robot bodyplans (43). We chose to evolve CPPNs using AFPO instead of HyperNEAT because the former is a much simpler algorithm than the latter. Despite the fact that it is more complex, it would be interesting to apply HyperNEAT to designing reconfigurable organisms; it could be that the additional machinery yields more performant designs.

Source code: github.com/skriegman/reconfigurable_organisms/blob/master/tools/algorithms.py

5.2. *Measuring performance.* In all experiments, designs are allowed to settle under gravity for one second before the evaluation period begins. Just before the evaluation period starts, the initial center of mass of the design is recorded as (x_0, y_0, z_0).

For locomotion, the performance score was net displacement of the design's center of mass, in terms of euclidean distance: the square root of $(x-x_0)^2 + (y-y_0)^2$, where (x, y) is the final position of the design on the ground plane at the end of an evaluation period of 10 seconds. For object manipulation (§10.1), transport (§10.2), and expulsion (§10.3), the object's net displacement was tracked instead of the design's.

It is important to note that the formulation of the performance objective function must in some cases be refined in order to realize desired behavior. For example, an early version of the objective function for object transport (Fig. S13) intended to reward how far an object could be carried. However, because the lightweight object never touched (i.e., penetrated) the simulated ground plane, the optimizer discovered designs that dragged the object along the ground plane. This was corrected by constraining the object to be completely surrounded by tissue (§10.2).

5.3. *Runtime.* Each evolutionary trial optimized a population of 51 designs on a dual-processor, 12-core Intel E5-2650 v4 (i.e., 24 threads). No trial took less than 18 wall-clock hours (432 CPU hours) or more than 22 wall-clock hours (528 CPU hours) to evaluate 1000 generations of evolutionary improvement. The runtime varies because the algorithm is stochastic: some designs have more voxels than others and thus require more CPU-time. Because evaluating designs in simulation is the computational bottleneck, the algorithm is readily parallelizable: doubling CPU threads halved the wall-clock time (10 hours when tested using two Intel E5-2650 v4s).

5.4. *Algorithm analysis.* It is difficult to know what the optimal design is for large search spaces. So, we investigated a search space in which it was possible for us, given our computational resources, to determine exactly what the optimal design is (for a given random actuation pattern). We started with a 2×2×2 workspace and identified the optimal design for five different, random actuation patterns (Fig. S17*A*). The evolutionary algorithm found the optimal design for all five actuation patterns in 9129 evaluations (179 generations). In the slightly larger, 3×2×2 workspace, the evolutionary algorithm found the optimal design for all five actuation patterns in 4284 evaluations (84 generations) (Fig. S17*B*). In a 3×3×2 workspace, the algorithm took much longer, requiring 113,628 evaluations (2228 generations) to find the optimal design for all five actuation patterns (Fig. S17*C*). We could not determine the optimal design at 3×3×3, so we terminated the determination of optimal designs at this point. Source code: github.com/skriegman/reconfigurable_organisms/blob/master/exp/Algorithm_Analysis_EA.py

5.5. *Comparison to a gradient-based approach.* We employed an evolutionary algorithm because we have considerable experience using this particular algorithm to evolve soft robots in previous work. However, there are many forms of constrained optimization. One of the most common is stochastic gradient descent. So, we applied a stochastic gradient-based method (Parameter-exploring policy gradients; (44)) directly to the design problem. Typically the policy that is optimized is a vector of neural network weights (floating-point values). Here, the policy is a static arrangement of the discrete building blocks (ternary values). The algorithm samples the space of designs, evaluates their performance *in silico*, and estimates the gradient using a popular stochastic gradient descent optimizer (Adam; (45)). This algorithm yielded a less performant design (4.6 body lengths per minute; Fig. S16) that failed all three conditionals of the build filter (§7.2). Source code, which was adapted from (46), is available here: github.com/skriegman/reconfigurable_organisms/blob/master/exp/Algorithm_Analysis_SGD.py

The same population size as the evolutionary algorithm was used; all other hyperparameters were left at their default values as reported in (46). The policy vector of floating-point values was discretized to determine tissue type at each point in the workspace: values below -0.05 were

left empty, values above 0.05 were encoded as passive voxels, and values in between -0.05 and 0.05 were encoded as contractile voxels. The two thresholds of -0.05 and 0.05 were set such that randomly-initialized designs at the beginning of optimization contained all three tissue types (the initial standard deviation is 0.10 by default).

This gradient-based algorithm quickly converges to suboptimal designs, even in very small search spaces (Fig. S17). To combat this premature convergence, we modified the algorithm to restart, every 1000 evaluations, from a different random initialization.

After 10,000 evaluations of search in the 2×2×2 workspace, the policy gradient algorithm found the optimal design for two of the five, random actuation patterns (40%). By restarting every 1000 evaluations, the modified algorithm was able to find the optimal design for four out of the five actuation patterns (80%) (Fig. S17*A*). In the 3×2×2 workspace, the algorithm did not find the optimal design for any of the five actuation patterns (0%). Modified to restart every 1000 evaluations, the algorithm found the optimal design for one of the five actuation patterns (20%) (Fig. S17*B*). In the 3×3×2 workspace, we allowed optimization to continue for much longer (140,000 evaluations), but the optimal design was not found for any of the five actuation patterns, with or without restarts (Fig. S17*C*). Source code: github.com/skriegman/reconfigurable_organisms/blob/master/exp/Algorithm_Analysis_SGDre.py

6. Updating design constraints.

This subsection describes how the design process is improved with feedback from the behavior of manufactured organisms.

On the first pass through the pipeline using the goal behavior of locomotion, the simulated environment consisted of an infinite plane and a gravitational acceleration of -9.81 m/s^2. Both the passive and contractile building blocks had a Young's modulus of 10^7 Pa, a Poisson's Ratio of 0.35, and coefficients of static and dynamic friction of 1.0 and 0.5, respectively. Source code: github.com/skriegman/reconfigurable_organisms/blob/master/exp/Locomotion_pass1.py

The contractile voxels were volumetrically actuated (±50% rest volume) at 5 Hz. Although the cardiac tissue used to build reconfigurable organisms can only contract, simulated actuation also expanded voxels in volume because this produces more force, and thus faster locomoting designs *in silico*. We chose to call these voxels "contractile" to help clarify the match with contractile tissue. Future design-manufacture cycles of the pipeline could add a contraction-only actuation constraint *in silico*, however it was not necessary for successful transferral of behavior (§9.1).

The phase-offsets of actuation for each voxel (from a global, sinusoidal signal) were co-optimized with morphology, using two independent genotype networks (§4.1). Designs evolved propagating waves of volumetric actuation, yielding rapid locomotion via bounding gaits, with timeframes (on average, 47% of the gait cycle) in which no part of the design was in contact with the simulated ground plane (Fig. S2). However, when these designs were manufactured *in vivo*, they always kept part of their ventral surfaces in contact with the surface of the dish due to negative buoyancy.

This discrepancy was rectified by adding the following constraints to the simulated environment and actuation. On the second pass through the design pipeline, an aqueous environment was simulated by decreasing the gravitational acceleration to -0.1 m/s^2, and

applying a drag force to surface voxels, assuming a fluid density of water (rho=1000 kg/m^3) and a drag coefficient of C=1.5 for each exposed voxel face.

Voxels were simulated with half the Young's modulus (5×10^6 Pa) and five times the length scale of those used during the first pass of the pipeline. This allowed a larger numerical integration timestep to be stable (see (22) for details), which greatly reduced the required CPU time of each evaluation.

Actuation frequency was reduced to 2 Hz to remove momentum effects (which are difficult to simulate accurately) and to better match the contraction rate of the cardiac tissue (~1 Hz). The phase-offset of each voxel was randomized instead of optimized, which prevents designs from overfitting to a specific actuation policy. Each randomly-configured design injected into the population (with age zero; see §5.1) was assigned 448 phase-offsets, randomly drawn from a uniform distribution between $-\pi/2$ and $\pi/2$, one value for every point (possible voxel location) in the 8×8×7 workspace. These phase-offsets were then hardcoded for the entire clade.

Evolutionary improvement within a clade thus occurred through changes in overall shape, and distribution of the passive and contractile voxels, to collectively derandomize the global movement produced by the random actuation. This reduced the dependence on precisely-timed actuation, which increased the likelihood of successful transferal from *silico* to *vivo*. Source code: github.com/skriegman/reconfigurable_organisms/blob/master/exp/Locomotion_pass2.py

The surface contact behavior of designs generated on the second pass (Fig. S3) better matched that of the actual living systems: On the second pass through the design pipeline, run champions were, on average, in contact with the ground plane for 93.3% of their evaluation period, compared to just 52.7% on the first pass. The proportion of simulation time that designs are in contact with the ground was computed by recording (100 times per second) whether or not the z position of any voxels were penetrating the surface plane, using the following source code: github.com/skriegman/reconfigurable_organisms/blob/master/data_analysis/Time_in_contact_with_ground.py

7. Filtering evolved designs for manufacture.

This subsection describes how designs were filtered based on their performance *in silico*, transfer potential, manufacturability under the current build method, and their scalability to more complex tasks in future deployments.

7.1. *The robustness filter.* The most performant designs (Fig. S3) were sorted by their robustness to random perturbations in their actuation (Fig. S4). Each design was then copied 20 times. The phase-offsets of the actuating voxels in each copy were independently mutated by adding a number that was drawn randomly (with random seeds 1-20) from a normal distribution with mean zero and standard deviation $s = 0.4\pi$ (which is 40% of the $-\pi/2$ to $\pi/2$ range of valid phase-offset values). This hyperparameter was selected to be large enough to scramble the original phase-offset value without being so large as to push all mutations up against the $\pm\pi/2$ bounds. Designs that maintained the highest average performance across this actuation noise were passed, one by one, in order of their robustness ranking, to the build filter. Source code: github.com/skriegman/reconfigurable_organisms/blob/master/data_analysis/Robustness_Filter.py

7.2. *The build filter.* The most robust designs were evaluated by their manufacturability under the current build method, which layers contiguous tissue regions sequentially, one on top of the other, with each layer filling the x,y plane (Fig. S6; §8.2). Thus, the first criterion a design must meet for fabrication is that it must contain contiguous tissue regions that fill the dorsal and ventralmost x,y planes with just one of the two tissue types (i.e., no mixing within the dorsal and ventral layers). The design can be rotated in 3D space to satisfy this criterion (e.g., without mixing within the anterior and posteriormost layers).

Secondly, designs cannot contain arbitrarily small gaps in their geometry, because they are made of differentiating cells which will adhere to neighbors if they come into contact with each other. The minimal concavity was examined by producing organisms with progressively smaller shape deformations, then determining which persist across the lifespan of the organism, and which close due to tissue adherence/contraction, leading to loss of concavity. Preliminary work determined that concavities with a width of 100μm or greater produced stable long term deformations suitable for biological building (Fig. S7). As the organisms typically have body diameters in the range 750±100μm at the time of initial cutting and 850±100μm after four days of healing, the minimal concavity width is 12%-14% of total body length.

Because larger designs can travel farther in the same amount of time as smaller ones, the *in silico* designs tend to fill at least one horizontal dimension of the 8×8×7 workspace (Fig. S3), resulting in a maximum length of 8 voxels. This equates to a minimal concavity width of a single voxel (1/8=12.5%). Thus, the second criterion for fabrication is that the design cannot contain gaps less than two voxels wide.

Finally, more complex tasks can require room for payloads (Fig. S13; §10.2) or, in future, the addition of specialized cells for purposes other than locomotion, including sensory input, metabolism, memory, biosensors, etc. Also, contractile tissue incurs a high metabolic cost (compared to non-muscle tissue), which decreases the total lifetime of transferred designs. Thus, the third criterion for fabrication is that the design must be mostly (more than 50%) passive. Source code: github.com/skriegman/reconfigurable_organisms/blob/master/data_analysis/Build_Filter.py

8. Manufacturing reconfigurable organisms.

This subsection summarizes the current build method.

8.1. *Cilia-driven organisms.* During preliminary experiments aimed at testing the feasibility of *in vivo* designs, the manufactured organisms lacked contractile tissue and were instead propelled by cilia present on the surface of the body. These cilia-propelled spheres were manufactured in exactly the same way as the cardiomyocyte-driven organisms used to measure transfer success (§8.2), except the Notch intracellular domain (Notch-ICD) was not overexpressed through synthetic mRNA microinjection (this step inhibits multiciliated cell formation) prior to building. However, all of the ciliated organisms moved when released into the aqueous environment (albeit in unpredictable directions and at unpredictable speeds). Thus, measuring transferability between *in vivo* and *in silico* designs could produce false positives. In addition, the accurate modeling of swimming and fluid dynamics proved to be challenging in simulation, so we altered our build method to produce contractile based movement.

8.2. *Cardiomyocyte-driven organisms.* Contractile organisms were generated using two separate approaches (detailed in Methods and Materials). In the first, presumptive cardiomyocyte and epidermal cells were extracted from embryos and dissociated in calcium free magnesium free medium. Dissociated cells were then transferred to a 1mm depression and the tissues were layered according to the desired *in silico* design. After two days of further development, final shaping was performed using a microcautery device and surgical forceps. For the second build method, the presumptive cardiomyocyte and epidermal tissues were not dissociated to the individual cell level. Instead, layers of tissue were stacked on top of one another, with the cardiomyocyte layers in the center. Shaping occurred in exactly the same manner, using a combination of microcautery and surgical forceps.

Both the dissociation and tissue layering method were employed during the course of the study, however, we chose to focus on the latter for movement-based assays. While this method results in lower accuracy of tissue placement (as specific numbers of cells could not be layered with precision), it significantly reduces total build time, allowing the investigator to produce approximately ten times the amount of organisms per unit time compared to the dissociation method.

9. Measuring transfer success.

This subsection explains how the behavior and structure of manufactured organisms were compared to those of the *in silico* design.

9.1. *Transferal of behavior.* The design was evaluated 25 times, resulting in 25 movement trajectories. Each time, the evolved set of phase-offsets for the actuating voxels was different, resulting in slightly different behavior (Fig. 4). This was done by adding to each of the original phase-offsets, a value that was drawn randomly from a normal distribution with mean zero and standard deviation $s = 0.4\pi$ (which is 40% of the $-\pi/2$ to $\pi/2$ range of valid phase-offset values). This hyperparameter was selected to be large enough to scramble the original phase-offset value without being so large as to push all mutations up against the $\pm\pi/2$ bounds.

The numerical integration step size was 0.0032 seconds. For each random actuation pattern, the design was allowed to settle under gravity for 312 timesteps (one second), and then evaluated for 18750 additional timesteps (60 seconds), resulting in a total simulation time of 61 seconds.

Six reconfigurable organisms were built which embodied this design. They were imaged live for 10 minutes in 0.75x MMR at 20°C using a Nikon SMZ-1500 microscope equipped with both top and substage illumination. Videos were captured using a Sony IMX234 at a sample rate of 30fps. Behavioral trajectories were extracted using Noldus Ethovision 14 software. Each trajectory was then smoothed using a one-dimensional gaussian filter with a kernel standard deviation of 30 seconds.

Statistical analysis. In measuring transfer success, we made three comparisons (controlling for false discovery rate; (47)):

1. movement heading *in vivo* relative to *in silico*;
2. net displacement *in vivo* upright relative to inverted; and
3. net displacement *in silico* upright relative to inverted.

For movement heading, the data consist of the dichotomous outcomes: either the designed organism moved in the predicted direction, or not. Although organisms could move in any direction (0 to 360° relative to the predicted heading) we discretize the space into four directions of possible movement (forward, backward, left, right), only one of which is considered a success (forward). We have six realizations of the design *in vivo* (six separate organisms) that were each reset to the origin four times. As resets to the origin are clearly not independent observations, n = 6. That is, we have six independent Bernoulli trials with probability of success q. Because there are four directions of possible movement, the null hypothesis is that organisms move in the predicted direction with probability q = 1/4. All instances moved in the predicted direction, though one organism (and its four resets) was more or less sessile (with small amounts of movement in the predicted direction). The most conservative use of the data is to consider only five of the six realizations to be successful, and thus $p = 4.6\times10^{-3}$. Controlling for false discovery rate (47), the null hypothesis is rejected at the 0.01 level of significance.

For net displacement, the data consist of paired replicates: anatomically-upright ("pretreatment") and anatomically-inverted ("posttreatment") observations from the same individual design. We are concerned with a shift in location (i.e., the median of the distribution of net displacement) due to inverting the design (the application of the "treatment"). We use a distribution-free signed-rank test (Wilcoxon). The null hypothesis asserts that each of the distributions for the differences (posttreatment minus pretreatment observations) is symmetrically distributed about 0, corresponding to no shift in location due to the treatment (inverting the design). *In silico*, $p = 9.7\times10^{-5}$. Controlling for false discovery rate, the null hypothesis is rejected at the 0.001 level of significance.

In vivo, four organisms were evaluated five times while upright, and five times inverted. However, the recording equipment failed during one of the upright runs (Trial 2) for three of the organisms. This trial was simply removed from consideration for these three (i.e., displacement while inverted in Trial 2 was discarded where corrupted). The two other organisms were evaluated five times upright, but only once while inverted since these organisms generated little to no displacement when inverted, and motion tracking resources were limited. The single evaluation while inverted was therefore used as the posttreatment observation for each of the six upright (pretreatment) observations. *In vivo*, $p = 2.6\times10^{-5}$. Controlling for false discovery rate, the null hypothesis is rejected at the 0.0001 level of significance.

In fact, only one of the six organisms was observed to produce appreciable forward movement while inverted. This anomaly was likely due to the cardiac tissue being layered deeper (more dorsally) than the other designs, resulting in a small amount of deformation on the dorsal surface.

Source code for reproducing the *in silico* behavioral trajectories: github.com/skriegman/reconfigurable_organisms/blob/master/data_analysis/Transferal_from_silico_to_vivo.py

Source code for the statistical analysis: github.com/skriegman/reconfigurable_organisms/blob/master/data_analysis/Statistical_Analysis.py

9.2. *Transferal of structure.* The structure of organisms was compared to that of the *in silico* design using 2D images and Hausdorff distance.

To quantify the structural error (Fig. S8), lineage labeled organisms were created by harvesting tdTomato expressing cardiac progenitor tissue from one set of donor embryos, and GFPIII expressing passive epidermis tissue from a second set of donor embryos. Donor tissue

was dissociated and transferred to 1mm concave wells, placing both cell types in proportions and locations matching the *in silico* design. Two days later, the organism was then sculpted to the desired shape, and imaged in multiple orientations using a FITC filter set to visualize epidermal tissue (green) and a TRITC filter set to visualize cardiac progenitor (red) tissue. Channels were then overlaid in ImageJ to create the final composite image for analysis. K-means clustering was used to classify each pixel as one of three tissue types: contractile, passive, or none.

The Hausdorff distance between *in vivo* and *in silico* pixels was calculated for passive tissue only, then again for contractile tissue. The closest matching pixel (i.e., with the same tissue type) was found *in silico* for all *in vivo* pixels, and the closest matching pixel was likewise found *in vivo* for all *in silico* pixels. The Hausdorff distance is the largest such discrepancy between *vivo* and *silico* tissue coordinates, in terms of euclidean distance in microns. A small Hausdorff distance indicates that for every pixel *in vivo* there is a pixel of the same tissue type nearby *in silico*, and vice versa. After measuring the Hausdorff distance for both tissue types, the larger of the two is taken to be the structural error.

Formally, the Hausdorff distance for tissue type 1 is defined as:

$$H_1 = \max \{ \sup_{s \in S_1} \inf_{v \in V_1} d(s, v), \ \sup_{v \in V_1} \inf_{s \in S_1} d(s, v) \},$$

where S_1 and V_1 are the sets of *in silico* and *in vivo* pixels that were classified as the first tissue type, and $d(s, v)$ is the euclidean distance between pixels s and v. Similarly, the Hausdorff distance for tissue type 2 is defined as:

$$H_2 = \max \{ \sup_{s \in S_2} \inf_{v \in V_2} d(s, v), \ \sup_{v \in V_2} \inf_{s \in S_2} d(s, v) \},$$

where S_2 and V_2 are the sets of *in silico* and *in vivo* pixels that were classified as the second tissue type. The structural error is then:

$$\max (H_1, H_2).$$

Before these comparisons can be made, however, the *in vivo* and *in silico* images need to be on a relatively consistent coordinate frame. Thus, images were auto-cropped such that the edge of the design fills the frame. This is done by converting each image to grayscale, and thresholding at 10/255 to create a binary image, thus isolating the organism against the dark background. Contours are then automatically drawn on the image by traversing the boundaries of each transition between black and white to find closed loops (each of which is a contour). A bounding box is drawn around the largest contour (by area), and the rest of the image is trimmed off. Finally, the cropped images were resized (downsampled) to a constant resolution (50×50).

A grid search was then performed to find the 3D perspective with the lowest structural error. The design was lowered/raised in elevation angle in the z plane, and spun around by azimuth angle in the x,y plane, in increments of 10° in each dimension. Then the 3D plot was saved as a 2D image and rotated again in increments of 10°.

Since the tissue regions are classified via unsupervised clustering (and were thus assigned arbitrary labels), we take the regions with the largest overlap to be of the same type. This introduces the possibility of similarly shaped but different tissue types aligning to achieve the lowest structural error for a given *in vivo* perspective, but this did not occur in our experiments. However, the rotation with the lowest structural error did not always respect the organism's anteroposterior alignment (the design was sometimes "facing" the opposite direction). So we restricted rotations of the design to better match the range of perspectives captured across the four images of the organism: the grid search was constrained to elevation angles between -40 and

60°, azimuth angles between -120 and -60°, and rotations of the resulting 2D image between 0 and 30°.

The average structural error achieved across all four images was 323 microns (38% of the organism's largest diameter) (Fig. S8).

Note that this preliminary method does not account for distortions resulting from flattening a 3D object to a 2D image (e.g., the moon terminator illusion). Future work will aim to capture many images of the organism from different perspectives, and use them to virtually reconstruct a 3D model for direct structural comparison. Source code: github.com/skriegman/reconfigurable_organisms/blob/master/data_analysis/Structural_Error.py

10. Applications.

This subsection summarizes four additional goal behaviors in which organisms can interact with an external object or other organisms.

10.1. *Object manipulation.* Introducing a single object to the environment, and altering the goal function to track the external object, instead of the design, yielded object manipulation—block pushing—*in silico* (Fig. S12). The new behavioral goal input into the pipeline was to maximize displacement of a 2×2×2 voxel object during an evaluation period of 30 seconds. This extended evaluation time of 30 seconds (instead of 10 sec) prevented the strategy of simply falling onto the object and hitting it forward ahead of the (often immobile) design. All other constraints from the second pass for locomotion were left in place: passive and randomly-actuating building blocks were reconfigured within an 8×8×7 workspace, and evaluated in a floored aqueous environment. Sixteen independent evolutionary runs were performed. In some designs, a primitive end-effector—a notch in the corner of the body—evolved to hold and manipulate the object as it was pushed along the floor. Source code: github.com/skriegman/reconfigurable_organisms/blob/master/exp/Object_Manipulation.py

10.2. *Object transport.* Some designs evolved for locomotion were hollow and could thus, in theory, be exapted to internally store the 2×2×2 voxel object as cargo, rather than push it externally. This realization led to the new goal of maximizing the euclidean distance of a carried object. Using a slightly larger, 10×10×9 workspace, a mask was placed on the design requiring the morphology to house the 2×2×2 object within a 4×4×4 voxel pouch. This yielded evolved object transport *in silico* (Fig. S13). All other constraints were identical to the second pass for locomotion (the evaluation period was reverted back to 10 sec). Sixteen evolutionary runs were performed.

Some modifications of the objective function were required to realize object transport. An early version of the objective function did not use masking to force the object to be inside the design at the center of the workspace. The object was instead free to be positioned anywhere in the workspace. Designs were then rewarded by net object displacement, with the stipulation that the evaluation period would be terminated if and when the object was detected to penetrate the floor. This is a standard way to ensure that a simulated object is touching the ground. However, given the buoyancy of the aqueous environment, the lightweight object would often touch but not penetrate the ground. What evolved were mostly variants that pushed or dragged the object

across the ground plane. Source code:
github.com/skriegman/reconfigurable_organisms/blob/master/exp/Object_Transport.py

10.3. *Object expulsion.* A non-locomotion-based goal was also supplied to the pipeline: maximize thrown object distance. In other words, evolve a catapult (48). We dropped a 2×2×2 voxel object (the projectile) from one voxel length above the 8×8×7 workspace, but no designs evolved to catch and throw the object forward. So we reverted actuation to be finely tuned (rather than randomized) by co-evolving phase-offset alongside structure, as in the first pass for locomotion. But, the evolved designs were relatively compact and lacked limbs for proper throwing. So, we induced direct selection pressure for limbs by incorporating an additional objective that maximized surface-area to volume ratio. In three of five evolutionary trials, limbs evolved which the design employed to throw the object (Fig. S14). However, all of the designs capable of throwing objects, failed to pass through the build filter (conditional B1 of the flowchart in Fig. S4) because they were composed entirely of muscle. Additionally, these designs rely on finely-tuned actuation, which is unlikely to transfer from *silico* to *vivo*. Source code: github.com/skriegman/reconfigurable_organisms/blob/master/exp/Object_Expulsion.py

10.4. *Collective behavior.* Multiple designs can be placed in a single environment instead of a single one. This can be done by simply relaxing the constraint which takes only the largest connected component to be the design. Alternatively, individually evolved designs can be evaluated together at a later stage in the pipeline. The latter method was implemented: five of the fastest locomoting designs (Fig. S3) were placed in the same environment amongst a grid of particulate matter in the form of 2×2×1 voxel particles, yielding spontaneous collective behavior and particle aggregation *in silico* (Fig. S10). Source code:
github.com/skriegman/reconfigurable_organisms/blob/master/exp/Collective_Behavior.py

Spontaneous collective behavior and aggregation of external particles also occurred *in vivo* when multiple (10 to 15) organisms were placed together at the center of a single petri dish containing carmine dye (Sigma-Aldrich C1022-5G) (Fig. S11). A stock solution of carmine dye was created at a concentration of 0.01g per 10ml 0.75x MMR and vortexed for 10 seconds. Individual working solutions were then created in 1% agarose coated polystyrene petri dishes by diluting the stock 1:10, again in 0.75x MMR, for a final concentration of 0.001g per 10ml. Dishes containing the working solution were housed under an imaging microscope and allowed to settle for four hours at 22°C, creating a layer of particulate dye on the surface of the dish.

As a control, organisms were withheld from the dish, and the dye did not self aggregate after 1 or 24 hours.

11. Scaling the pipeline.

This subsection describes how future design-manufacture pipelines may improve to scale the complexity and competence of reconfigurable organisms.

In the work reported here, actuation *in silico* was constrained to be random (§6) because it is not yet understood how to model the dynamics of cells in novel configurations and environments. This proved sufficient for some open-loop tasks, but not for others which required finer control (§10.3). Before a general understanding of the relationship between cell signaling

and behavior is realized, behavioral biases of cells, or correlations between cells in certain configurations could be captured and distilled down into new constraints. However, in order to accurately incorporate the constraints of more realistic dynamics, the resolution of the simulation might need to be improved in future experiment designs.

Designs were constrained to 8-by-8-by-7 resolution (448 voxels) but the cardiomyocyte-driven organisms contained ~5,000 cells (Fig. S18). The resolution of the workspace could be increased, but the required CPU-time scales with $O(n+s^2)$, where n is the total number of voxels, and s is the number of voxels that lie on the surface of the body, which are used to detect and resolve collisions. Such computational bottlenecks can be circumvented by incorporating an additional generator-filter pair along the pipeline that draws individuals from an evolving population of low-resolution designs (and/or low-resolution task environments) to evaluate at higher-resolution. In effect, the low-resolution population would serve as a computationally-cheaper surrogate model for higher-resolution designs and environments. However, it is currently not known how to guarantee that behavior generated in low resolution design space can be preserved when mapped onto higher resolution spaces.

At the time of writing, a GPU-accelerated version of the physics engine was released (creativemachineslab.com/titan-library) which can purportedly simulate millions of voxels in near real time. This raises the possibility of simulating reconfigurable organisms at the cell level, and exporting the design directly to a 3D bioprinting/cell-printing device containing multiple dispensers (each loaded with a bio-ink of an individual cell type), which could in principle exactly recapitulate the design *in vivo*. However, much work remains to adapt existing technologies for this purpose.

Supplementary References

36. The Online Encyclopedia of Integer Sequences (nos. A000162 and A001931). https://oeis.org/A001931
37. K. O. Stanley, Compositional pattern producing networks: a novel abstraction of development. *Genetic programming and evolvable machines* **8**, 131-162 (2007).
38. J. Clune, B. Beckmann, C. Ofria, R. Pennock, Evolving coordinated quadruped gaits with the HyperNEAT generative encoding. *Proceedings of the IEEE Congress on Evolutionary Computation* (2009).
39. J. Auerbach, J. Bongard, Environmental Influence on the Evolution of Morphological Complexity in Machines. *PLoS Computational Biology*, **10**, e1003399 (2014).
40. T. Elsken, J. Metzen, F. Hutter, Neural Architecture Search: A Survey. *Journal of Machine Learning Research*, **20**, 1-21 (2019).
41. K. Stanley, R. Miikkulainen, Evolving Neural Networks through Augmenting Topologies. *Evolutionary Computation*, **10**, 99-127 (2002).
42. K. Stanley, D. D'Ambrosio, J. Gauci, A Hypercube-Based Encoding for Evolving Large-Scale Neural Networks. *Artificial Life*, **15**, 185-212 (2009).
43. N. Cheney, R. MacCurdy, J. Clune, H. Lipson, Unshackling Evolution: Evolving Soft Robots with Multiple Materials and a Powerful Generative Encoding. *Proceedings of the 15th Annual Conference on Genetic and Evolutionary Computation*, 167-174 (2013).
44. F. Sehnke, C. Osendorfer, T. Rückstieß, A. Graves, J. Peters, J. Schmidhuber, Parameter-exploring policy gradients. *Neural Networks*, **23**, 551-559 (2010).
45. D. Kingma, J. Ba, Adam: A method for stochastic optimization. *arXiv preprint arXiv:1412.6980* (2014).
46. D. Ha, Evolving Stable Strategies (2017). blog.otoro.net/2017/11/12/evolving-stable-strategies
47. Y. Benjamini, Y. Hochberg, Controlling the false discovery rate: a practical and powerful approach to multiple testing. *Journal of the Royal Statistical Society: Series B (Methodological)*, **57**, 289-300 (1995).
48. N. Chaumont, R. Egli, C. Adami, Evolving virtual creatures and catapults. *Artificial life*, **13**, 139-157 (2007).

Video S1: Designing reconfigurable organisms.

Link: **youtu.be/VVd_MjHm_tc**

Video S1 first previews successful output of the pipeline: the champion design of run 52 *in silico* and its realization *in vivo*. Next, the input—the goal (move into the left-hand side of the screen, as fast as possible) and building blocks (passive and contractile voxels)—are introduced. The building blocks are shown in pairs (two passive, cyan blocks are connected and positioned on the floor next to a separate pair of contractile, red/green blocks) to better illustrate the actuation dynamics. At 0:30, three random configurations of the building blocks are shown in action. These three configurations were the best designs (of 50 random designs) found during generation zero of three independent evolutionary runs with goal behavior of net displacement. One thousand generations later, the most robust design to pass through the build filter—the champion of run 52—is selected for construction *in vivo*. At 1:14, video of the virtual design (top) is complemented by video of its realization *in vivo*: a computationally-designed organism locomoting with contractile muscle in an aqueous environment (presented at 4x speed). At 1:27, an organism is shown to move in the direction predicted by the in-silico model (one second of video corresponds to 1.25 real-time minutes). At 1:37, the organism is rotated 180° about its transverse plane (placed on its back) and fails to generate forward movement. At 2:12, the collective behavior of multiple designs were deployed in an aqueous environment and filmed in timelapse. Individuals are observed forming stable duplets and triplets, changing partners across the observation. This behavior was predicted and observed by the in-silico model (particularly at 2:20). At 2:28, the organism is deployed into an aqueous environment with an agarose substrate covered in carmine particles, and spontaneously pushes one of the particles forward through a circling movement pattern. A design explicitly evolved *in silico* for pushing a particle (yellow) is also shown for comparison. At 2:39, a design evolved for locomotion is exapted for object transport (yellow). Following a demonstration of the brittleness of our current technologies, at 3:02, an organism was subjected to mechanical laceration with microsurgery forceps. Timelapse imaging demonstrates wound closure and repair over the course of 10 minutes.

Video S2: Manufacturing reconfigurable organisms.

Link: **youtu.be/kCOKtmmNH90**

Video S2 provides a timelapse recording of the entire build process for *in vivo* representations of the *in silico* designs. The process begins with the microinjection of fertilized *Xenopus* embryos (1.15-1.2mm in diameter) with synthetic mRNAs encoding lineage tracers (tdTomato [red] or GFPIII [green]) or proteins to alter cell fate. At 0:10, after developing for 24 at 14°C, the vitelline membrane is removed from each embryo with microsurgery forceps. At 0:23, the animal cap of each embryo is then surgically excised with forceps and incubated for 10 minutes in dissociation media. At 0:36, the outer ectodermal layer (which does not dissociate) is manually separated from the inner cell layers (approximately 30μm in diameter). The remaining tissue is gently agitated to further aid with dissociation, before being pooled with a micropipette (1:08). At 1:20, hundreds of cells and at 1:27, thousands of cells are transferred to agarose wells containing standard media (different cell types can be layer sequentially at this step), promoting cell re-aggregation. At 1:34, cell aggregates undergo compaction as they re-adhere and are allowed to develop for two additional days at 14°C. At 1:50, a microcautery device is utilized to define the rough shape of the transferred design (650μm-750μm in diameter) and microsurgery forceps are then used to sculpt the final features.

Table S1. Biological reagents.

1	*Xenopus* frogs and embryos:	Nasco LM00490, LM00531, LM00456
2	Standard Xenopus media (10x stock):	1M NaCl, 20mM KCl, 10mM $MgSO_4$, 20mM $CaCl_2$ dihydrate, 20mM HEPES, ph 7.4.
3	0.75x Xenobot media:	Diluted from 10x Stock Xenopus media
4	Cell dissociation media:	50.3mM NaCl, 0.7mM KCl, 9.2mM Na_2HPO_4, 0.9mM KH_2PO_4, 2.4mM$NaHCO_3$, 1.0mM EDTA, pH 7.3.
5	tdTomato lineage tracer:	Waldner C, Roose M, Ryffel GU. 2009. BMC Dev Bio. 9(1):37.
6	GFPIII lineage tracer:	Zernicka-Goetz M, et al. 1996. Development. 1;122(12):3719-24.
7	NotchICD cilia inhibiting construct:	Deblandre GA, et al. 1999. Development. 1;126(21):4715-28.
8	1% agarose substrate for cap movement trials:	VWR EM-2125
9	Petri Dish, 60mm x 15mm polystyrene:	Fisher FB0875713A
10	Carmine dye:	Sigma-Aldrich C1022-5G
11	Gentamicin:	ThermoFisher Scientific, 15710072

Table S2. Shaping tools.

1	Microsurgery forceps:	Dumont, 11241-30 #4
2	Forcep sharpening stone:	Fine Science Tools 29008-22
3	Microcautery device:	Protech International Inc., MC-2010
4	13 micron cautery electrode:	Protech International, 13-Y1
5	Binocular dissecting microscope:	VWR 19000-852

Table S3. Imaging.

1	Fluorescent microscope:	Nikon SMZ-1500
2	Microscope camera:	QImaging Retiga 2000R CCD
3	Ipod touch for time lapse imaging:	Apple Inc.
4	Ipod microscope mount:	Gosky Binocular adapter B013D2ULO6
5	ImageJ image analysis software:	National Institutes of Health
6	Motion tracking software:	Noldus Ethovision 14

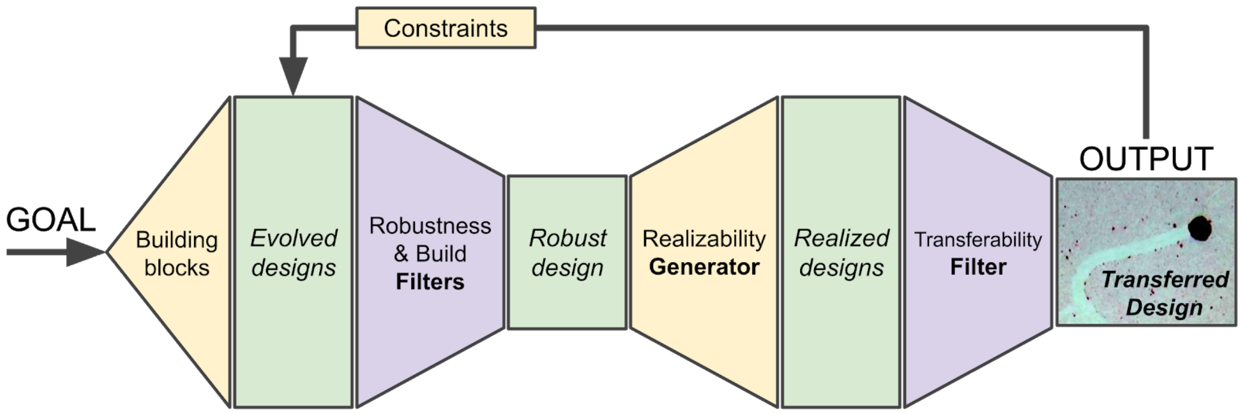

Fig. S1. Pipeline diagram. The pipeline goes from left to right—from input goal to in-silico design to in-vivo output, and back. The pipeline continues to loop indefinitely, or until some external termination condition is reached.

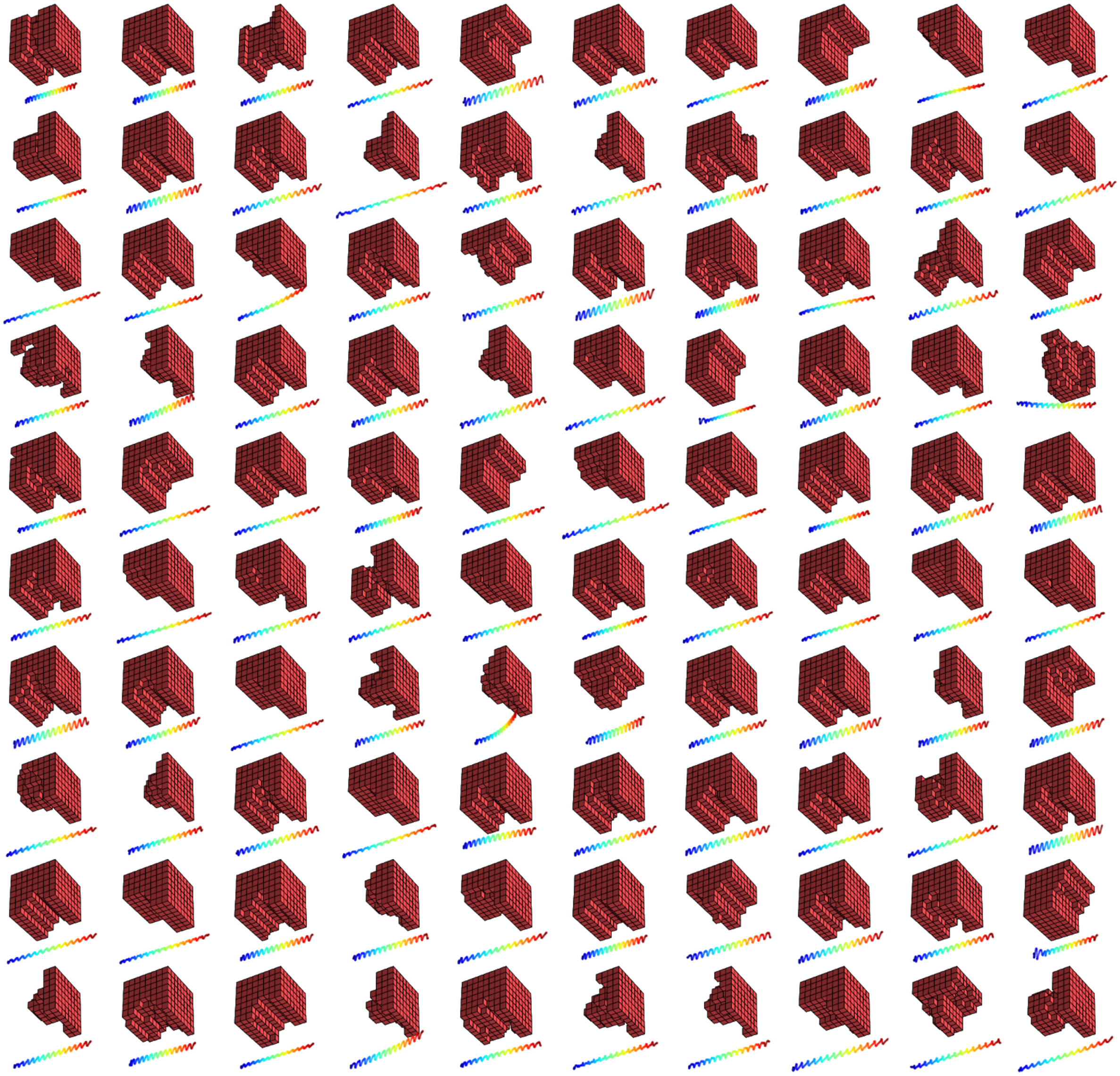

Fig. S2. The fastest designs from the first pass of the pipeline for locomotion. During the first pass of the pipeline for the goal behavior of locomotion, designs were evaluated in an Earth-like terrestrial environment and evolution was permitted to finely tune the phase-modulation (from a global signal) of actuation in each voxel. Under these conditions of precise actuation, and despite an objective explicitly minimizing the number of contractile voxels (red), the best designs (defined by their locomotive ability) did not utilize any passive material. There was also a strong convergence in shape: many independent runs converged to similar geometries. The rainbow streak below each design is its behavior—from initial (blue) to final position (red)—, superimposed, shifted and scaled to fit on the image (each streak is identically scaled). The run champions for runs 1 through 100 (with random seeds 1-100) are pictured in row-major order.

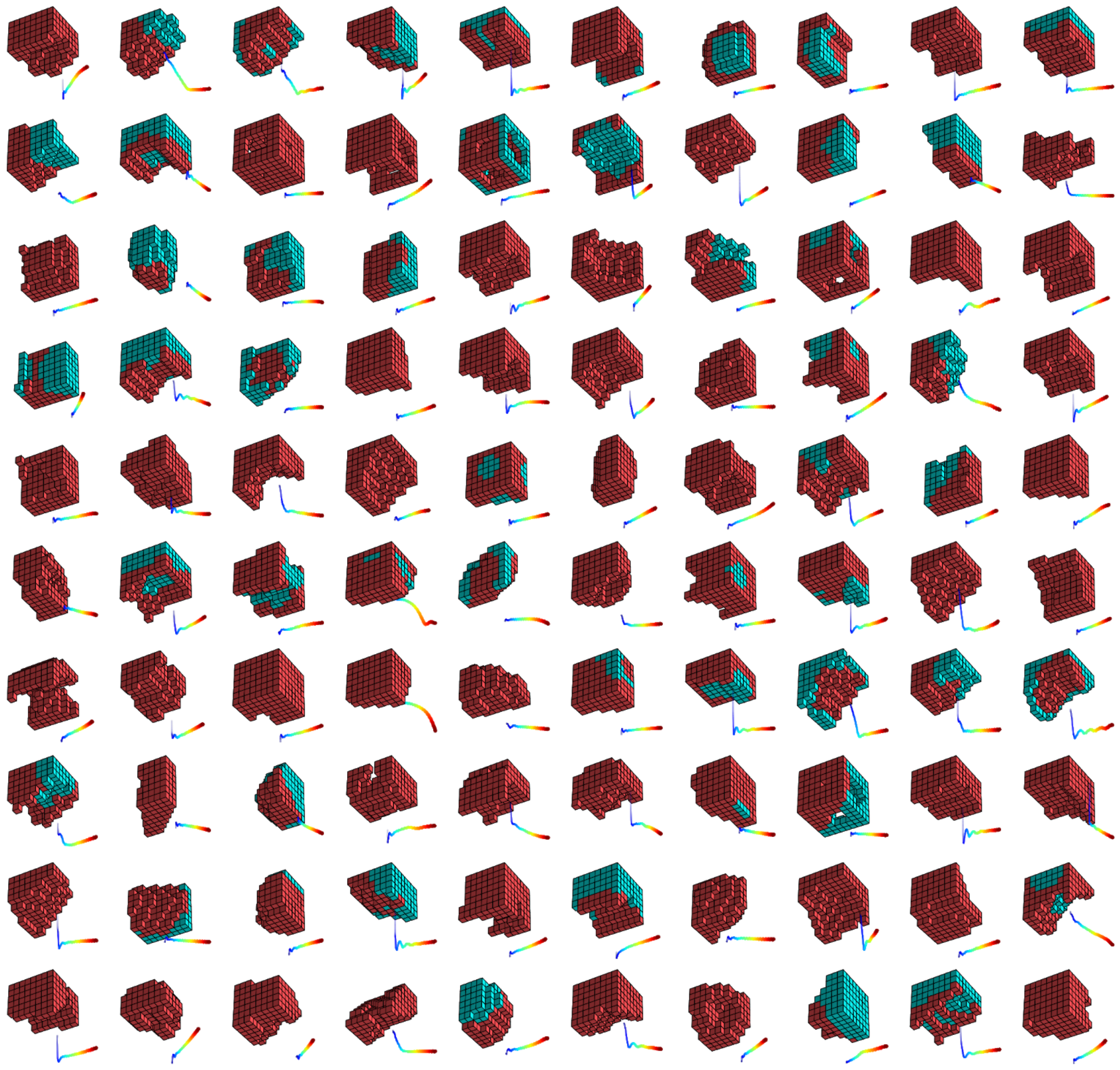

Fig. S3. The fastest designs from the second pass of the pipeline for locomotion. The second pass of the pipeline for locomotion incorporated a few additional constraints: First-order hydrodynamics were added, tissue density was decreased (made lighter), tissue elasticity was decreased (made softer), and actuation was randomized (each voxel has a randomly-assigned phase-offset which is fixed in all descendants). Because actuation is provided by fewer and less-coordinated active cells, the behavioral trajectories (rainbow streaks below the design) have smaller amplitude, and the final displacement was decreased compared to the first pass (Fig. S2). Although designs were allowed to settle under gravity for one second before the evaluation period (and actuation) begins, an appreciable portion of the evaluated behavior sometimes began with the design settling to the floor (falling blue portions of the rainbow curves). (Behavioral streaks are scaled identically within this figure, but different from those in Fig. S2.) These additional constraints increased geometric diversity, promoted the use of passive (cyan) tissue, and generated behavior that better matched that of the biological representations.

A. Robustness filter.

Avg. velocity rank	Run	Failed conditional
1	90	B1, B3
2	75	B3
3	43	B2, B3
4	5	B3
5	48	B1, B2, B3
6	69	B3
7	51	B2, B3
8	88	B2, B3
9	20	B2, B3
10	76	B2, B3
11	79	B2, B3
12	12	B1, B2
13	84	B1, B2
14	29	B2, B3
**15**	**52**	**None**
16	99	B1
...	...	...
100	31	B1

B. Build filter.

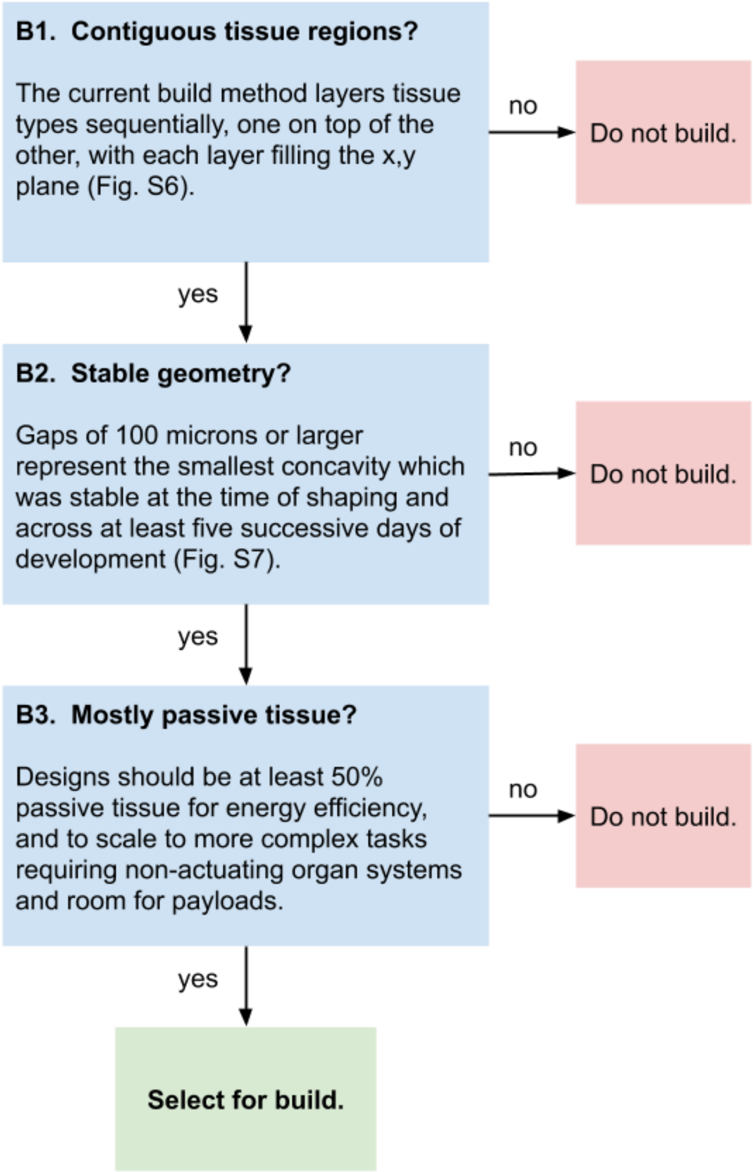

Fig. S4. The design filters and selection criteria. The most performant design from each evolutionary run (the 100 designs in Fig. S3) are filtered in order to select the most promising one (in terms of estimated transferability, buildability, and scalability) for manufacture. The most performant designs are first ranked by their median performance (velocity) under random actuation (robustness filter; *A*). Then, in rank order, each design is evaluated by the three conditionals (*B1*-*B3*) in the build filter (*B*), which removes designs that are not suitable for the current build method, and/or those which are not scalable to more complex tasks in future deployments.

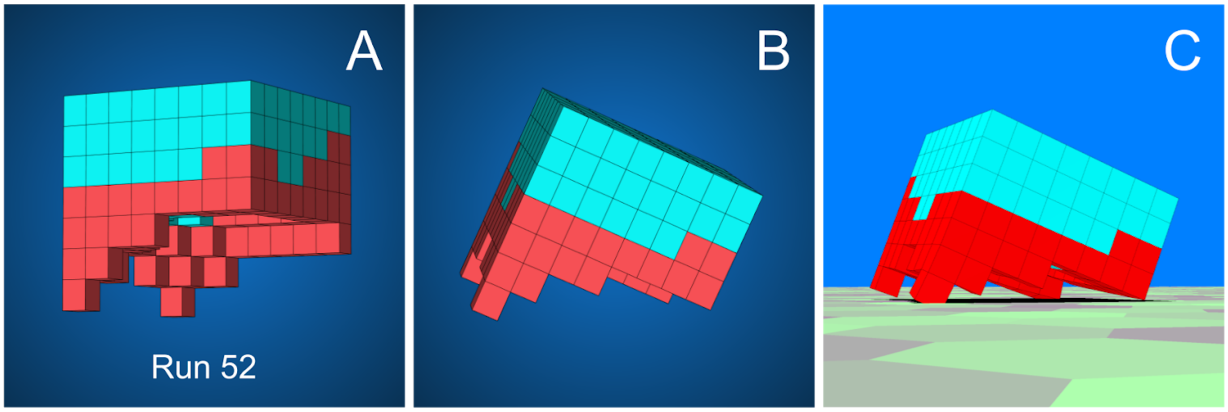

Fig. S5. The most robust design to pass through the build filter: The champion of run 52 (locomotion; second pass) is drawn floating (*A*, *B*), and resting on the surface in simulation (*C*).

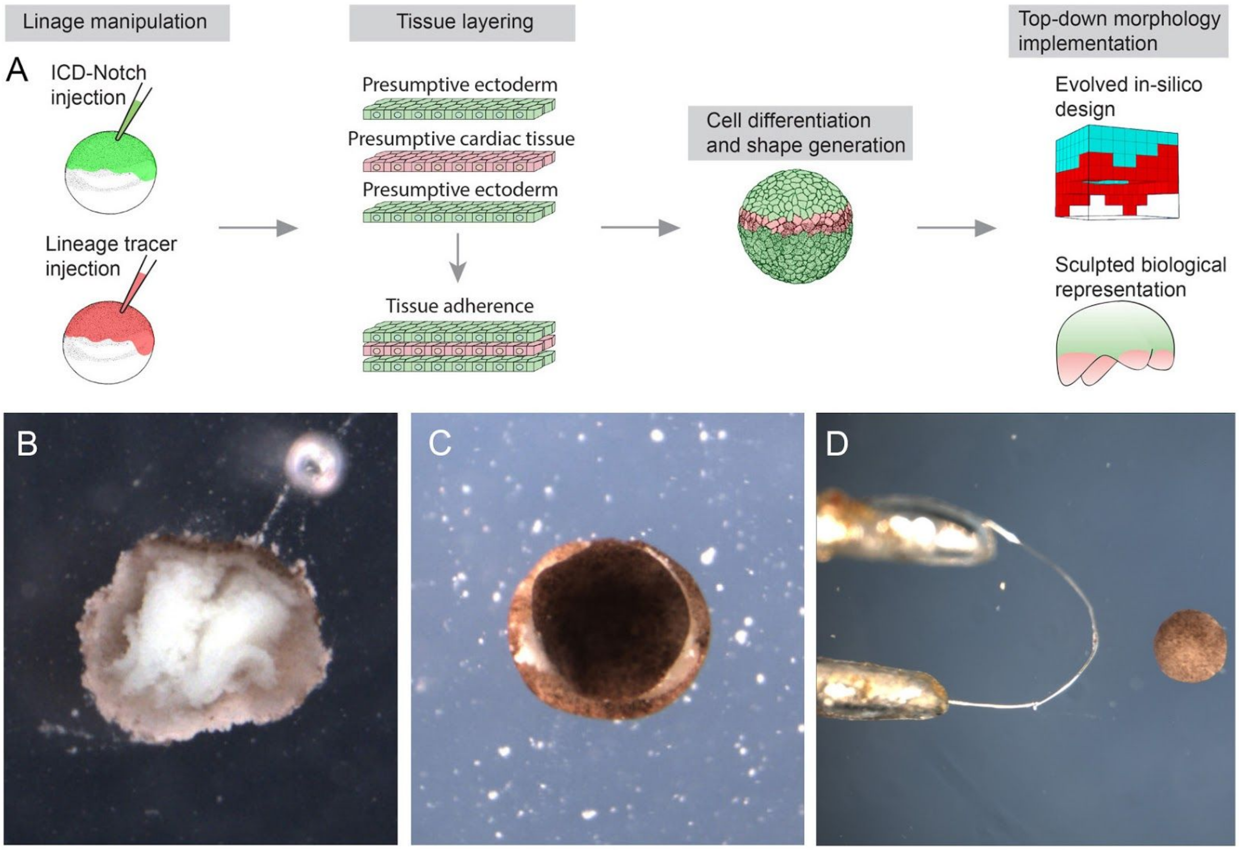

Fig. S6. Manufacturing cardiomyocyte-driven reconfigurable organisms. mRNA constructs are delivered to cleave stage *Xenopus* embryos which inhibit multi-ciliated epithelial cell differentiation and enable tracking of heart muscle tissue, allowing multiple cell types to be combined and intelligently shaped (*A*). Tissues are layered sequentially, first with an underlying layer of unspecified epithelium upon which cardiac progenitor tissue is deposited (*B*). A second layer of unspecified epithelium is layered and allowed to heal for an hour (*C*). Shaping is then applied to contractile regions using a microcautery electrode to produce the final shape (*D*).

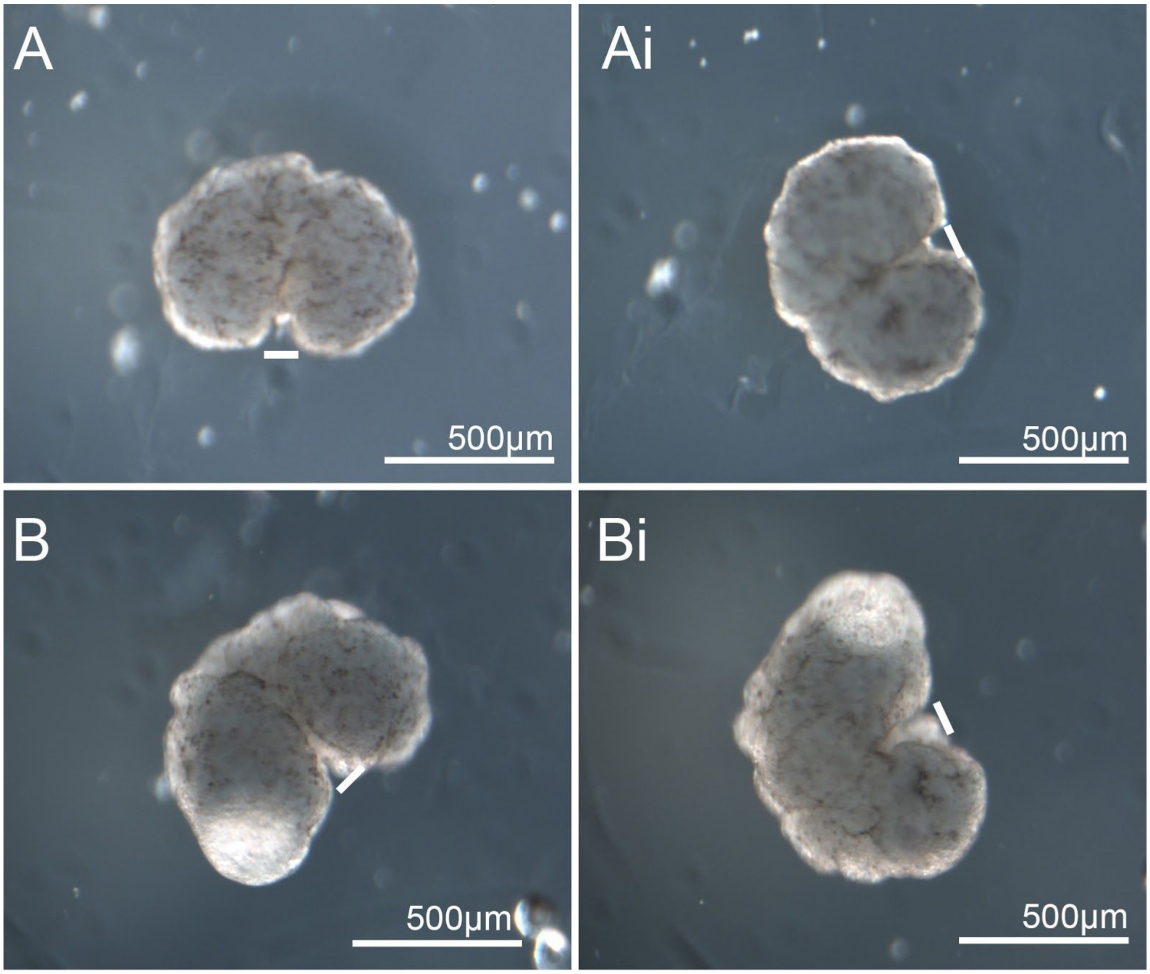

Fig. S7. Determining minimal concavity when shaping reconfigurable organisms. To optimize the build filter when selecting *in silico* designs for biological sculpting, spherical biobots were subjected to progressively smaller shaping gaps to evaluate the minimal size which persists across the organisms lifespan. Gaps of 100 microns or larger (white line) represented the smallest concavity which was stable at the time of shaping (*A*, *Ai*) and across at least five successive days of development (*B*, *Bi*).

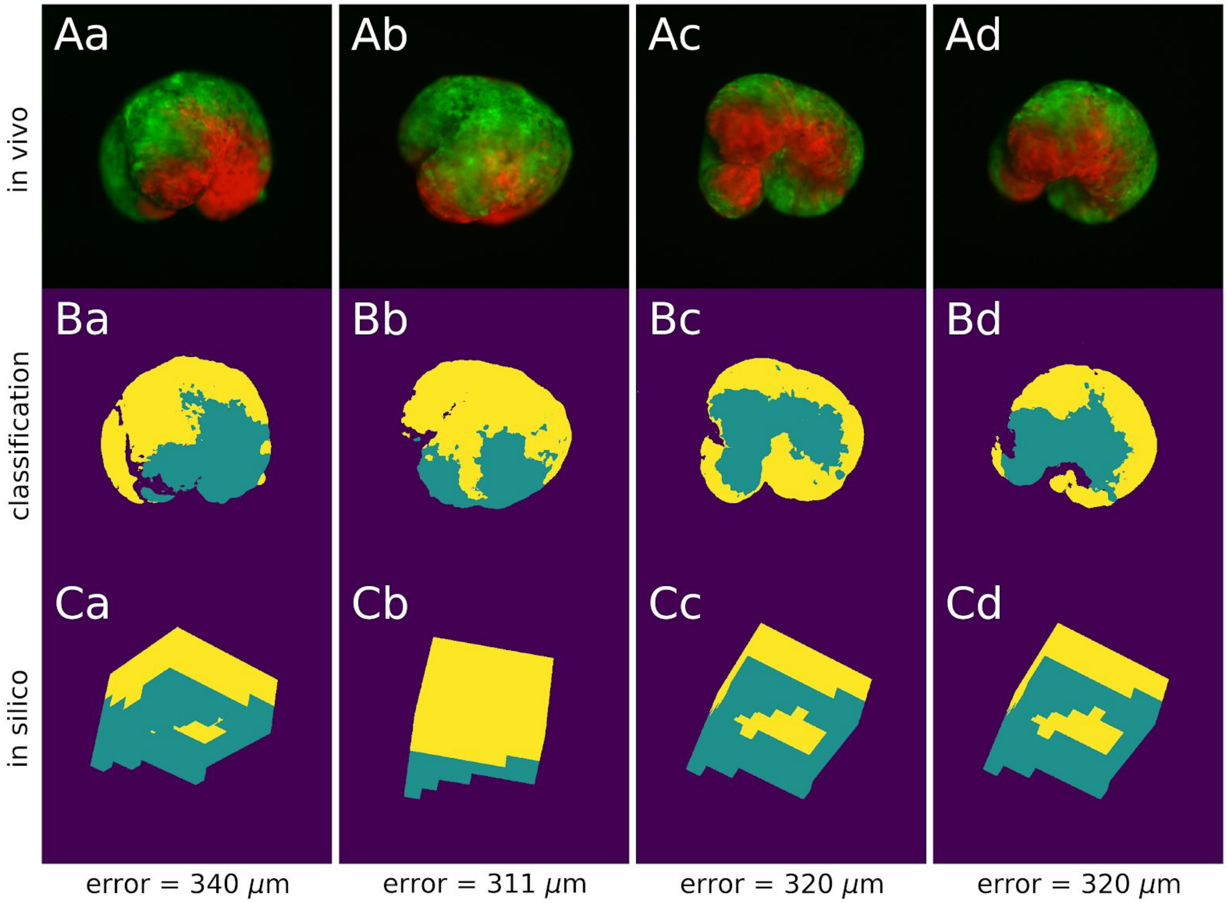

Fig. S8. Quantifying the structural match. Lineage labeled organisms were imaged in multiple orientations (*A*) using a FITC filter set to visualize epidermal tissue (green) and a TRITC filter set to visualize cardiac progenitor (red) tissue. K-means clustering was used to classify each pixel as one of three tissue types: contractile, passive, or none (*B*). The Hausdorff distance between *in vivo* and *in silico* pixels (*C*) was calculated for each tissue type separately, in terms of euclidean distance in microns. Overall structural error is taken to be the larger of the two Hausdorff distances, computed for the two tissue types. The design is automatically cropped and rotated in 3D space to find the 2D perspective with the smallest error.

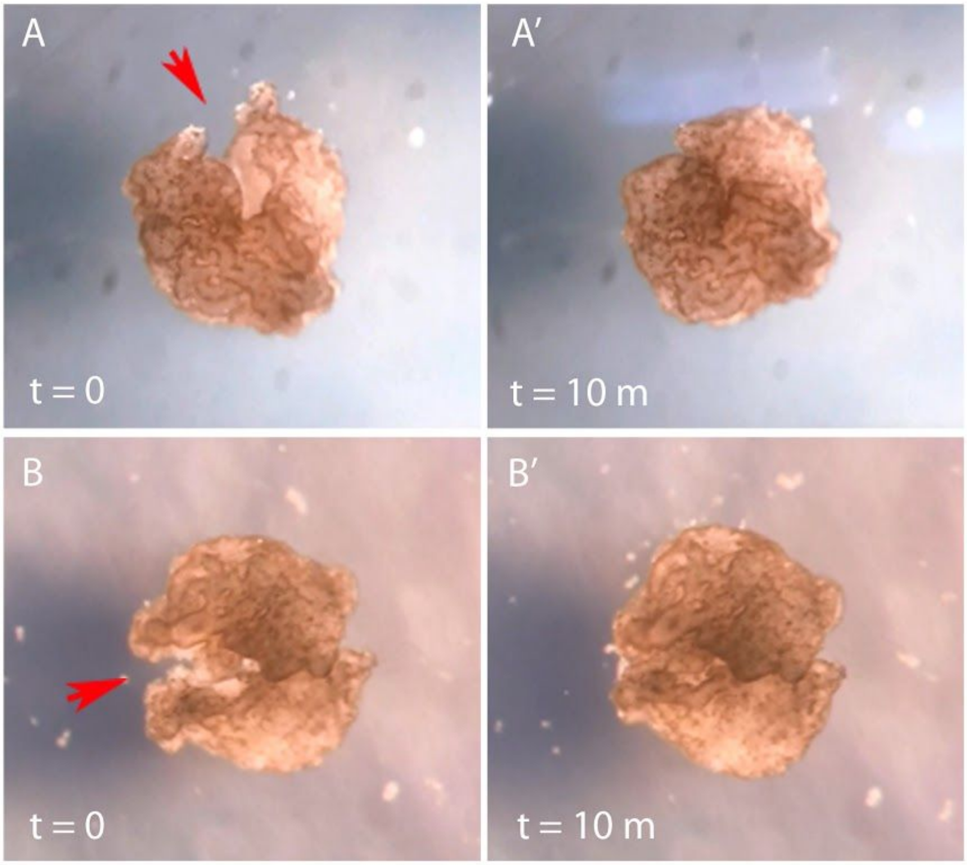

Fig. S9. Automatic self-repair in reconfigurable organisms. One week-old non-ciliated reconfigurable organisms were subjected to mechanical laceration with microsurgery forceps (*A*, *B*). Each individual was filmed with time lapse imaging, every five seconds, for 10 minutes. In all cases, the biological representations were able to close the wound and survive through the remainder of the experiment (*A'*, *B'*). Red arrows indicate the site of mechanical damage. Organisms were able to heal much larger wounds during repair experiments than during their initial shaping (Fig. S7). This is because shaping requires subtraction of tissue over a longer period (which physically inhibits the wound from shutting) while repair involves a single laceration delivered in a short duration. Also, in repair experiments, the organisms possess a fully developed epidermis (which is expected to close wounds) compared to shaping experiments where all cells are embryonically derived and are still in the process of differentiation.

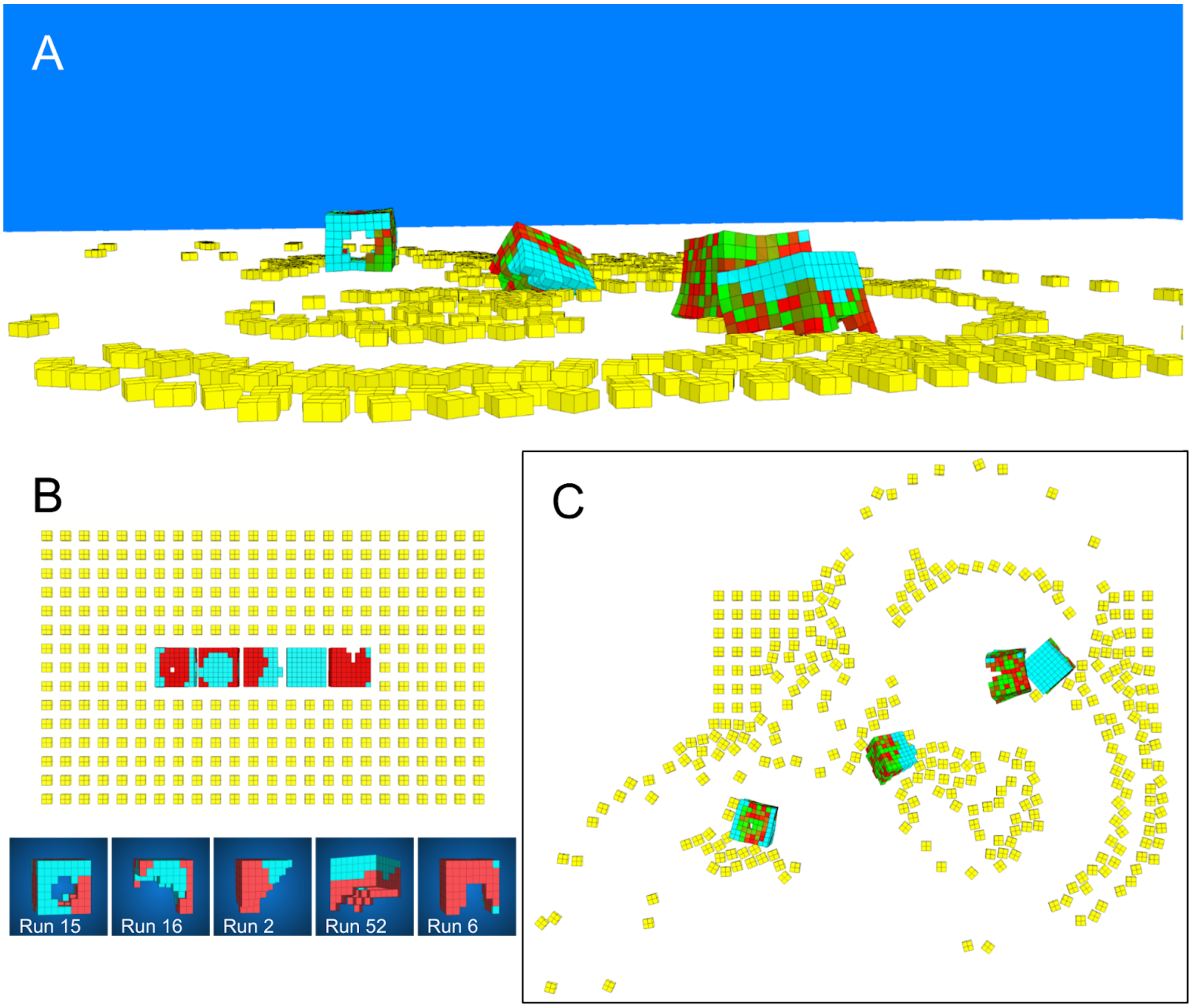

Fig. S10. Spontaneous collective behavior and particle aggregation *in silico*. Collective behavior of multiple designs interacting with yellow particulate matter in the form of 2×2×1 voxel particles (*A*). Five designs (locomotion run champions, from the second pass; Fig. S3) are initially placed amid a 15×15 lattice of the debris (*B*). One of the designs (run 16) travels relatively straight, while the other four move in elliptical orbits, often colliding and coupling for multiple revolutions along a new orbit before collision with a third causes them to detach along a transposed version of their soloist trajectory (*C*). There was no top-down selection for such connections, they occur spontaneously and affect the manner in which the duo interacts with the external objects. In terms of clearing debris, the performance of the interlocked pair is sometimes more efficient than the sum of its parts.

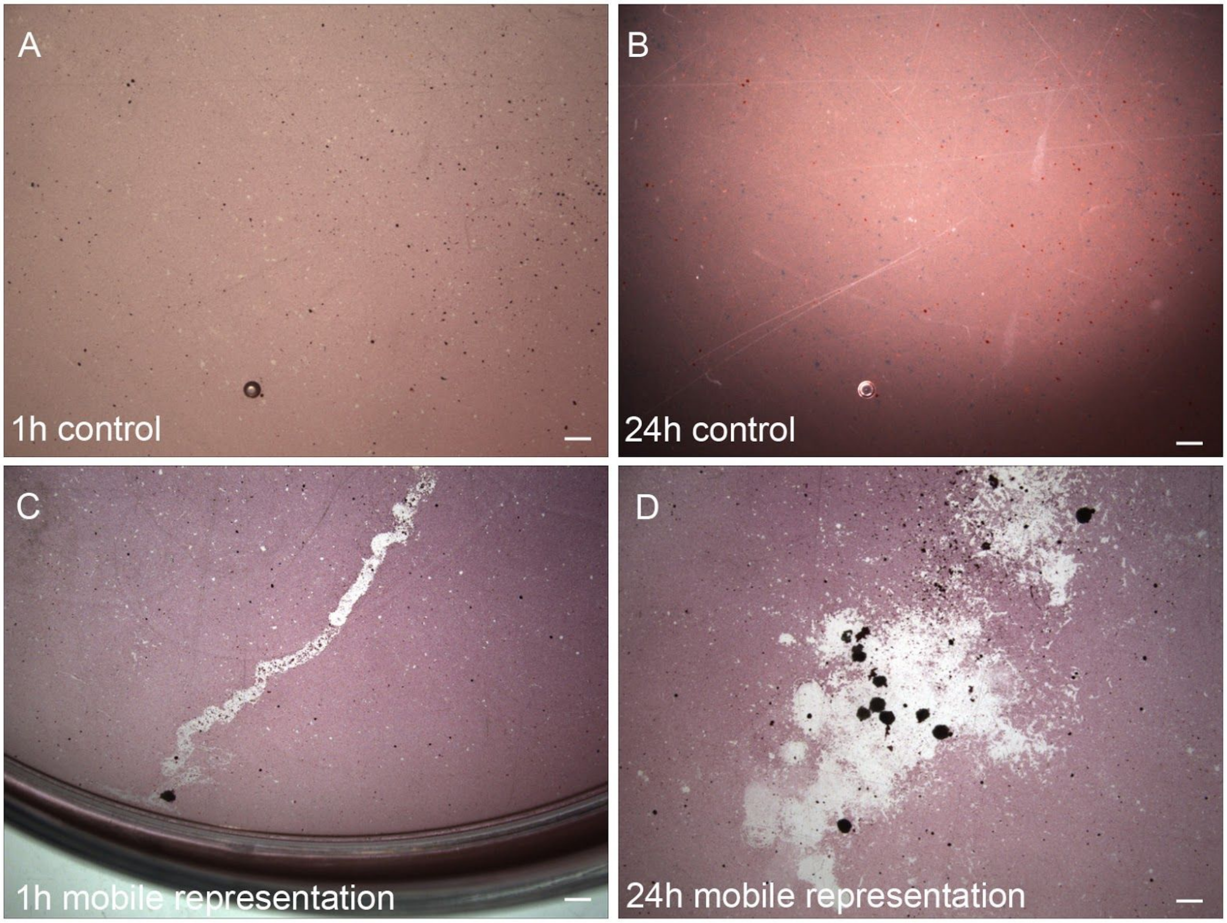

Fig. S11. Aggregation of carmine particles occurs only in the presence of organisms. In the absence of reconfigurable organisms, carmine dye does not self aggregate after 1 or 24 hours (*A*, *B*). However, in the presence of self locomoting organisms, movement of dye particles by individuals can be observed after one hour (*C*), and collective aggregation is observed by 24h (*D*). Scale bar indicates 1 mm.

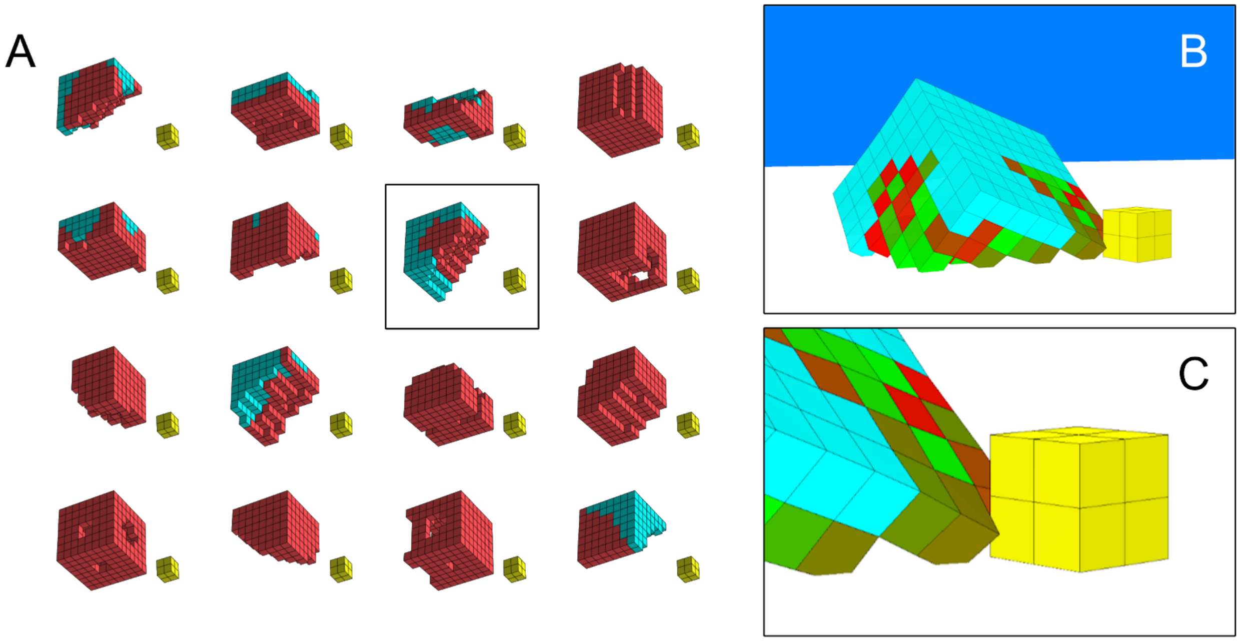

Fig. S12. Explicit selection for object manipulation *in silico*. A new goal was input into the pipeline: maximize displacement of a 2×2×2 voxel object (yellow), during an evaluation period of 30 sec. Sixteen independent evolutionary runs (with random seeds 1-16) produced sixteen run champs (*A*). In some designs, a primitive end-effector—a notch in the corner of the body—evolved to hold and manipulate the object (*B, C*).

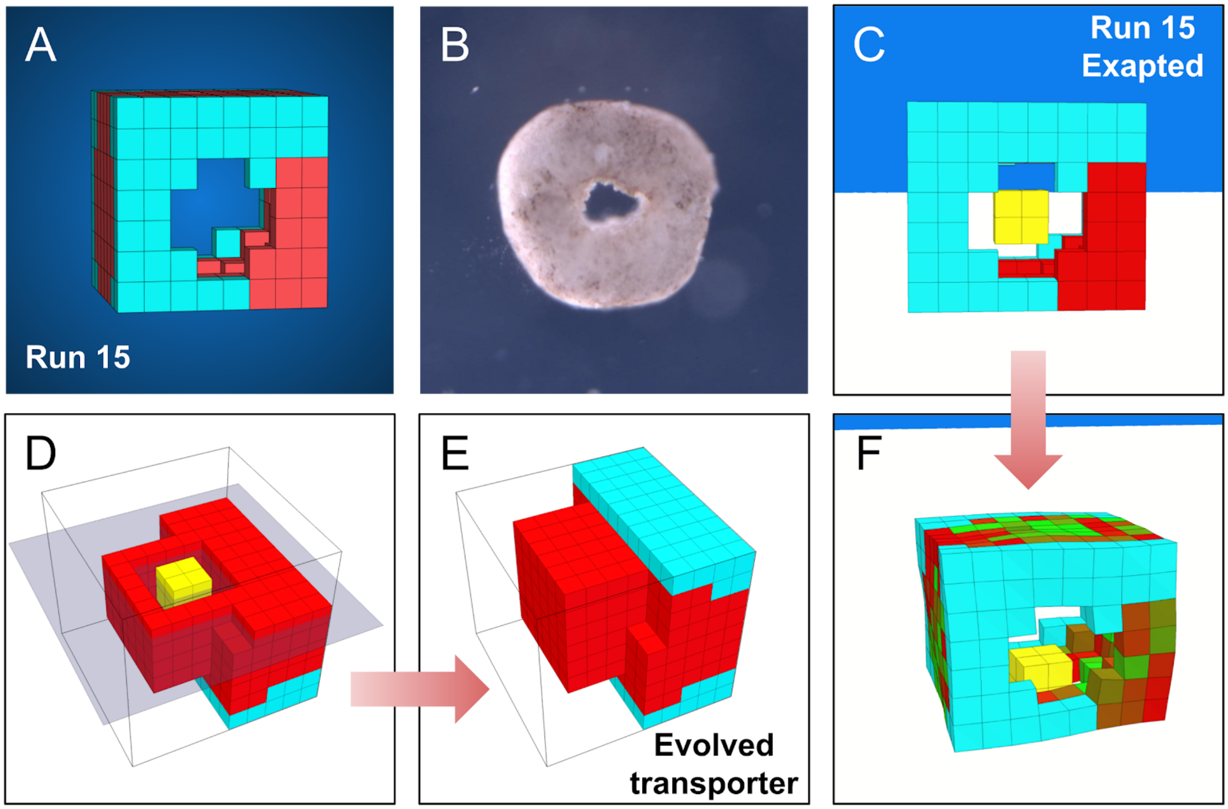

Fig. S13. From spontaneous to explicitly-optimized object transportation *in silico*. Some designs evolved for displacement reduced hydrodynamic drag via a hole through the center of their transverse plane (e.g., the champion of run 15; *A*). This more complex topology was realized *in vivo* (B) but was not layered with contractile tissue. In simulation, this emergent feature was exapted as a pouch to store and transport objects (the 2×2×2 yellow "pills" in *C*). This realization led to a reformulation of the goal behavior for a subsequent round of evolution, in which pouches—with a free-floating pill inside them—were explicitly incorporated as a design constraint (cross-sectional view in *D*), and the new goal of maximizing the euclidean distance of the carried object was employed. This yielded evolved object transport *in silico* (*E*). Sixteen runs were performed with this new constraint and objective. The run 8 champion (seed=8) at the end of 350 generations is shown in *D* and *E*.

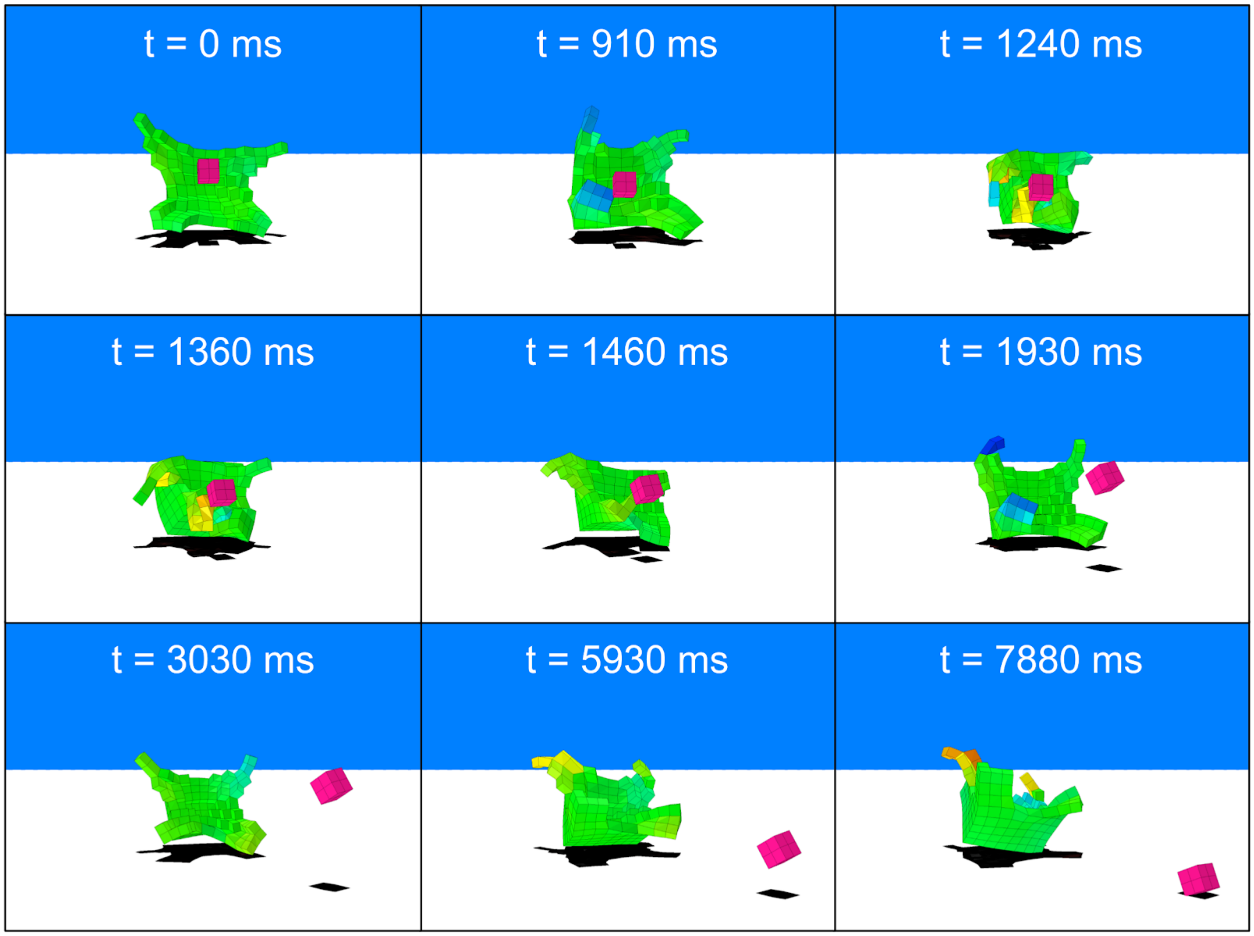

Fig. S14. Object expulsion *in silico*. A non-locomotion-based goal was supplied to the pipeline: thrown object distance (48). Using an additional objective that maximizes surface-area to volume ratio, limbs evolved which were used to throw a pink 2×2×2 object. For visibility, the voxels in this figure are colored by their angle relative to the ground plane, where green denotes parallel (angle zero), cooler colors (cyan and blue) denote voxel rotations into the right hand side (more or less) of the frame up to $-\pi$, and warmer colors (yellow, orange and red) denote voxel rotations into the left hand side up to $+\pi$. The run 2 champion (seed=2) at the end of 1000 generations is pictured. However, this design relies on finely-tuned actuation (which is unlikely to transfer) and fails to pass through the build filter because it is 100% muscle.

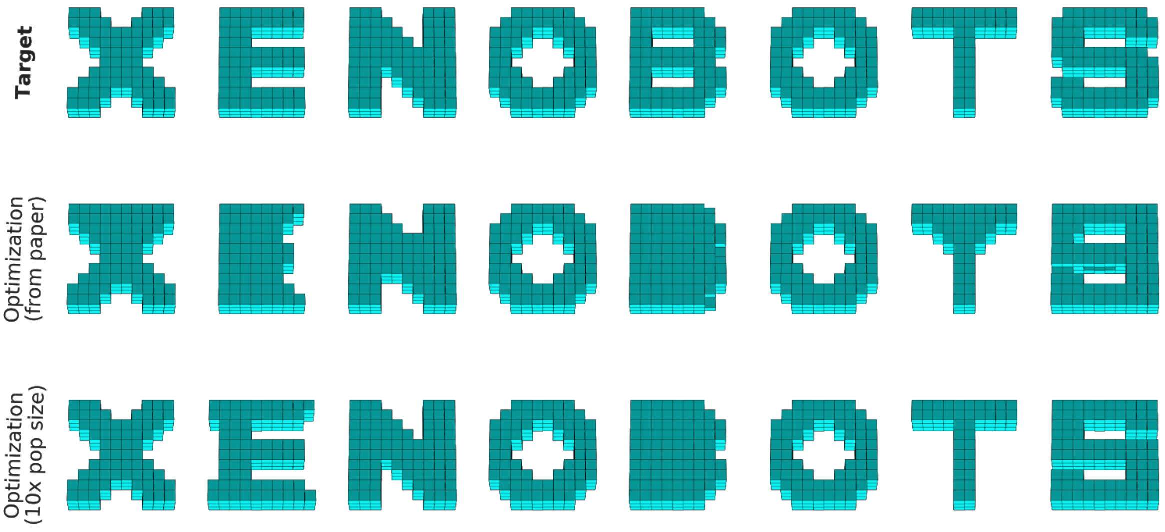

Fig. S15. The encoding bias. Explicit geometry optimization (passive tissue only) within a 10-by-10-by-3 workspace. We chose seven unique target shapes—the voxelyzed letters “X”, “E”, “N”, “O”, “B”, “T”, and “S”—and employed the same indirect encoding (CPPNs; (37)), optimization algorithm (AFPO; (19)), and hyperparameters as in the main paper. Each shape can be represented as a bitstring with length 300, where each element corresponds to the presence (1) or absence (0) of a voxel at one of the 300 cartesian coordinates in the workspace. The objective function measures the Hamming distance between a given shape and the target.

We re-optimized with a 10 times larger population size (500) and parallelized across 10 CPUs, which produced much more accurate solutions in the same wall-clock time (about 25 minutes per target). This suggests that shape (if not behavioral) complexity scales with population size.

The difficulty of realizing certain target shapes with this encoding, is due to the particular activation functions we used—sin(), abs(), square(), sqrt(abs())—which tend to produce patterns that gradually vary in space, and thus bias search away from abrupt perimeter changes, such as the square cutaways and holes in “E”, “B”, and “S”. This in-silico bias toward round edges mimics an in-vivo bias in our experiments: the pooled stem cells form a sphere during incubation, and naturally round-off abruptly carved edges after shaping.

In future design-manufacture cycles, if particular structures are known to be obtainable *in vivo* and useful for a given task environment (e.g., long appendages for reaching), mathematical functions that promote such features (e.g., a step function) can be included in the in-silico design.

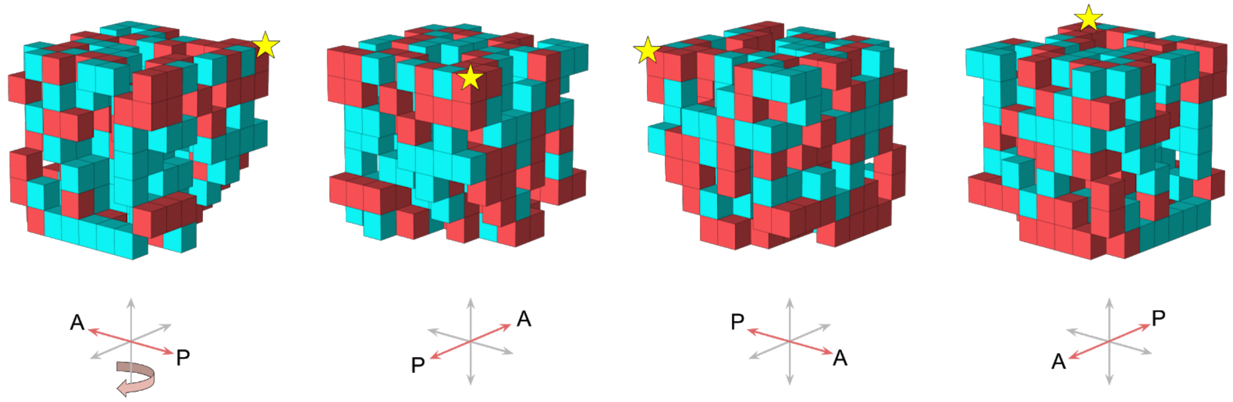

Fig. S16. Stochastic gradient descent directly applied to the design problem. A policy gradient method (44) was paired with a popular SGD optimizer (45) and directly applied to the design problem given a random actuation pattern (seed=1). The solution returned by this algorithm is shown from four different perspectives, with a yellow star drawn above one of the voxels for reference. It is less performant (4.6 body lengths / min) than the solutions found by the evolutionary algorithm. But more importantly, it is not manufacturable using the current build method: it fails all three conditionals of the build filter (Fig. S4).

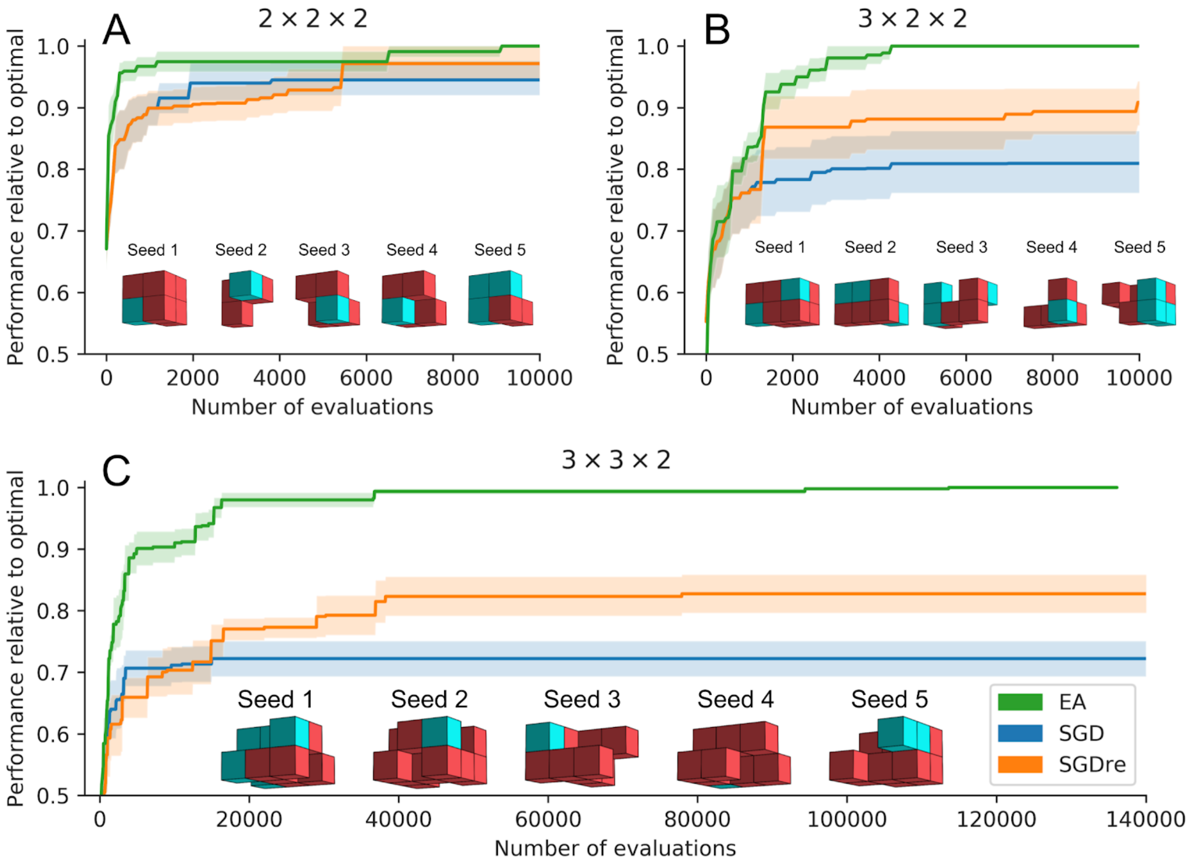

workspace	2x2x2	3x2x2	3x3x2	3x3x3	4x3x3	...	8x8x7
configurations	6,561	531,441	3.87×10^{8}	7.63×10^{12}	1.5×10^{17}	...	5.63×10^{213}

Fig. S17. Algorithm analysis. The optimal design was identified in small solution spaces for five different random actuation patterns (seeds 1-5). The optimal designs in each workspace (2×2×2, 3×2×2, and 3×3×2) are pictured under their corresponding random seed. The evolutionary algorithm used in this paper (green curves; EA) is plotted alongside a policy gradient method (44) which used stochastic gradient descent (blue curves; SGD). Because SGD tends to prematurely converge to a suboptimal design, the algorithm was then modified to restart search from a new random initialization, every 1000 evaluations (orange curves; SGDre). The mean performance of each algorithm (relative to that of the optimal solution) is shown as a solid line. The shaded areas around the lines are bootstrapped 68% confidence intervals of the mean (which for normally distributed random variables, corresponds to plus/minus one standard deviation). The table at the bottom lists the total number of configurations in each workspace, not accounting for isomorphisms, such as translations or certain rotations, or those which result from taking the largest connected component to be the design.

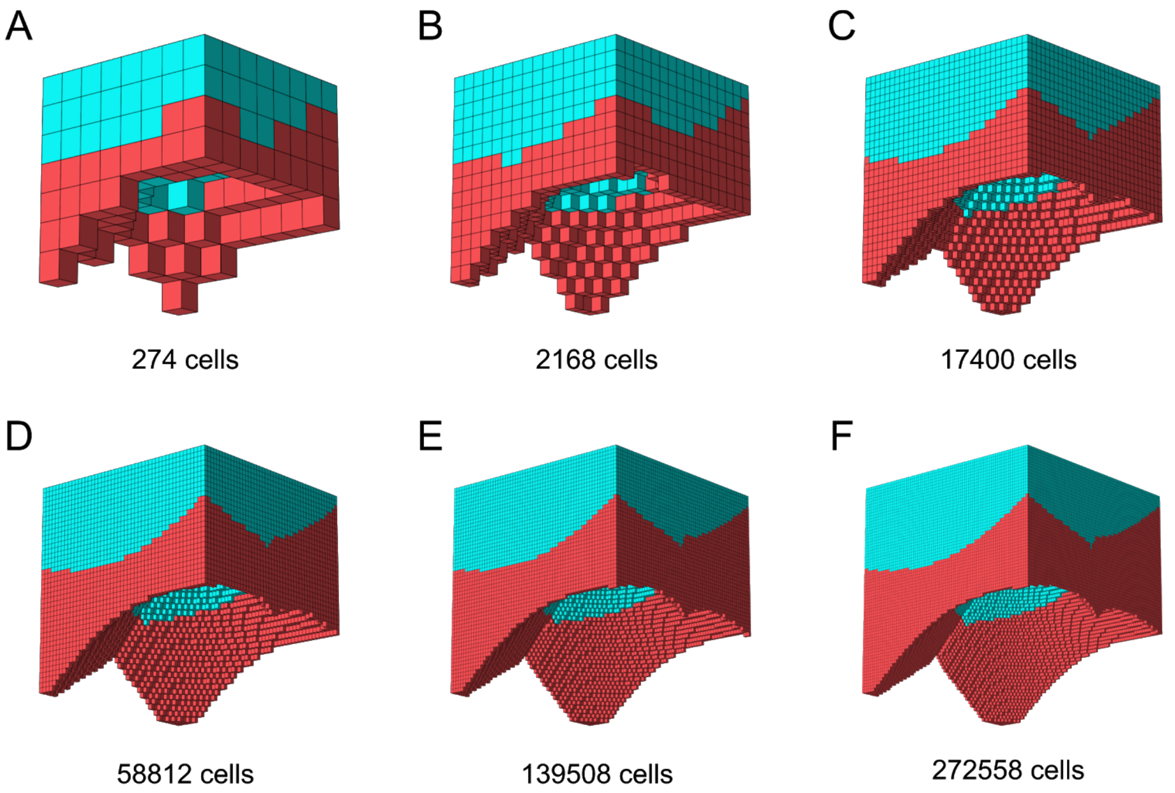

Fig. S18. Scaling the pipeline. Designs were "carved" *in silico* from 8-by-8-by-7 workspace (448 voxel "cells") but the organisms were carved *in vivo* from a 10000 cell sphere, and their final geometries contain about 5000 cells. However, because the genetic encoding is scale-free, evolved designs (*A*) can be scaled to a higher resolution *in silico* (*B-F*) while preserving geometry, but not necessarily behavior. To determine the behavior of anatomically-upscaled designs, additional simulations are required, the CPU-time of which increases with each virtual voxel. Thus upscaling phenotypes can significantly slow the pace of evolution. However, a GPU-accelerated version of the simulator was recently released which, by updating voxels in parallel, may alleviate this concern.
